# Drying of Soft Colloidal Films

**DOI:** 10.1002/advs.202406977

**Published:** 2024-11-05

**Authors:** Keumkyung Kuk, Julian Ringling, Kevin Gräff, Sebastian Hänsch, Virginia Carrasco‐Fadanelli, Andrey A. Rudov, Igor I. Potemkin, Regine von Klitzing, Ivo Buttinoni, Matthias Karg

**Affiliations:** ^1^ Institut für Physikalische Chemie I: Kolloide und Nanooptik Heinrich‐Heine‐Universität Düsseldorf Universitätsstr. 1 40225 Düsseldorf Germany; ^2^ Institute for Condensed Matter Physics Soft Matter at Interfaces Technische Universität Darmstadt Hochschulstr. 8 64289 Darmstadt Germany; ^3^ Center for Advanced Imaging Heinrich‐Heine‐Universität Düsseldorf Universitätsstr. 1 40225 Düsseldorf Germany; ^4^ Institut für Experimentelle Physik der kondensierten Materie Heinrich‐Heine‐Universität Düsseldorf Universitätsstr. 1 40225 Düsseldorf Germany; ^5^ DWI‐Leibniz Institute for Interactive Materials 52056 Aachen Germany; ^6^ Physics Department Lomonosov Moscow State University 119991 Moscow Russian Federation

**Keywords:** capillary forces, core‐shell microgels, fluid interface‐mediated colloidal assembly, microgel‐to‐substrate adhesion, thin film

## Abstract

Thin films made of deformable micro‐ and nano‐units, such as biological membranes, polymer interfaces, and particle‐laden liquid surfaces, exhibit a complex behavior during drying, with consequences for various applications like wound healing, coating technologies, and additive manufacturing. Studying the drying dynamics and structural changes of soft colloidal films thus holds the potential to yield valuable insights to achieve improvements for applications. In this study, interfacial monolayers of core‐shell (CS) microgels with varying degrees of softness are employed as model systems and to investigate their drying behavior on differently modified solid substrates (hydrophobic vs hydrophilic). By leveraging video microscopy, particle tracking, and thin film interference, this study shed light on the interplay between microgel adhesion to solid surfaces and the immersion capillary forces that arise in the thin liquid film. It is discovered that a dried replica of the interfacial microstructure can be more accurately achieved on a hydrophobic substrate relative to a hydrophilic one, particularly when employing softer colloids as opposed to harder counterparts. These observations are qualitatively supported by experiments with a thin film pressure balance which allows mimicking and controlling the drying process and by computer simulations with coarse‐grained models.

## Graphical abstract

The drying of soft colloidal films on solid interfaces is a complex process. In this study, core‐shell (CS) microgels are confined at the air/water interface, and investigated for the drying behavior on solid substrates using video microscopy. Variation in shell softness and substrate wettability reveal the complex interplay of different forces including capillary attraction and adhesion to the substrate influencing the microstructure.

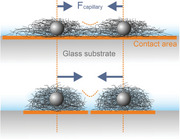

## 1 Introduction

Surface‐active agents are ubiquitous in our daily lives and paramount for numerous industrial applications including emulsion stabilization, foam formation, detergency, pharmaceutical drug delivery, and structured surfaces.^[^ ^1^ ^]^ They often consist of amphiphilic molecules (surfactants) that adsorb onto the interface between two immiscible phases (e.g., water and air), lower the surface tension, and stabilize the interface, e.g., against droplet coalescence. Remarkably, the same stabilization can be achieved via adsorption of solid micro‐ and nanoparticles, as in the case of Pickering emulsions.^[^ ^2^ ^]^ Compared to molecular surfactants, solid particles are believed to offer enhanced stability over extended periods of time, potentially exhibit lower toxicity,^[^ ^3^ ^]^ and generate interfacial films with significantly greater thickness due to their larger size.^[^ ^4^ ^]^ Additionally, solid particles need longer times to adsorb (in line with their characteristic diffusion timescale). Their presence at the interfaces often leads to the deformation of the interface due to their weight/size,^[^ ^5^ ^]^ shape,^[^ ^6^ ^]^ porosity,^[^ ^7^ ^]^ and/or wetting properties,^[^ ^8^ ^]^ which give rise to capillary interactions among adjacent particles. For example, the attractive force between two particles at the air/water interface (flotation capillary force) increases with the sixth power of the particle size and becomes negligible when the particle is smaller than 10 µm.^[^ ^9^, ^10^ ^]^ Conversely, particles as small as protein globules can be subjected to the influence of a strong attractive force when trapped in a thin water film (immersion capillary force).^[^ ^11^ ^]^ The horizontal projection of the attractive (immersion) capillary force *F_x_* between two (solid) spheres of radius *R* at a center‐to‐center distance *D*_c‐c_ partially immersed in a liquid is given by:^[^ ^12^ ^]^

(1) $$F_{x\;}\approx 2\pi \sigma R_{c}^{2}\;\left(\mathrm{si}n^{2}\;\Psi_{c}\right)\left(\frac{1}{D_{c-c}}\right)$$

Here, σ is the surface tension, *R_c_* the radius of the three‐phase contact line at the particle surface and $\Psi_{c}$ is the mean meniscus slope angle.^[^ ^11^ ^]^ Even for submicrometer‐sized particles, the capillary attraction can be much larger than the thermal energy. Since the interaction force is directly proportional to σ, capillary attraction is particularly strong in the drying of aqueous films.

Interfacial deformations, that ultimately also dictate $\Psi_{c}$, have a dramatic impact on the way a film dries on rigid surfaces, potentially leading to highly inhomogeneous drying conditions during coating and deposition processes.^[^ ^13^, ^14^, ^15^ ^]^ Inhomogeneous drying is known from the segregation of solutes to the edge of drying droplets leading to a ring‐like deposit (“coffee ring effect”).^[^ ^16^ ^]^ This phenomenon is driven by outward fluid flow caused by non‐uniform evaporation flux.^[^ ^16^ ^]^ On the other hand, a gradient in interfacial tension in a liquid film leads to flow caused by the Marangoni effect.^[^ ^17^ ^]^ Understanding such effects that strongly influence the drying of colloidal films is crucial in many areas including printing, colloidal self‐assembly, and coating technologies. The problem of inhomogeneous drying can be potentially solved by introducing strong repulsion among the solid particles^[^ ^17^, ^18^ ^]^ or employing “sticky” and deformable polymeric chains/networks.^[^ ^19^, ^20^ ^]^ Examples are poly(*N*‐isopropylacrylamide) (PNIPAM) microgels, i.e., soft colloid‐like objects with an internal gel‐like structure composed of a highly swollen crosslinked polymer network.^[^ ^21^ ^]^ Through simple precipitation polymerization, such microgels can be synthesized with some control over their internal architecture, size, and softness. Much like their solid counterparts, PNIPAM‐based microgels also spontaneously adsorb at various interfaces, e.g., foams, foam films,^[^ ^22^ ^]^ and emulsions.^[^ ^23^, ^24^, ^25^, ^26^ ^]^ At these fluid interfaces microgels are stretched along the interfacial plane, assuming a “fried egg” shape.^[^ ^27^, ^28^ ^]^ At large enough packing fractions, microgels can be finally assembled into closed‐packed viscoelastic films of high porosity – properties that are reminiscent of biological interactions (e.g., cell adhesion^[^ ^29^, ^30^ ^]^) and interfaces.^[^ ^31^ ^]^ Such close‐packed microgel films can be used to prepare thermoresponsive, free‐standing membranes that allow for manipulating ion transport by temperature.^[^ ^32^ ^]^ Controlling the packing fraction of microgels at fluid interfaces has also been used to prepare ordered monolayers on rigid substrates that can be used to create complex tesselations via self‐templating assembly.^[^ ^33^ ^]^ Core‐shell (CS) microgels with plasmonic gold or silver nanoparticle cores can be used to fabricate plasmonic surface coatings.^[^ ^34^, ^35^ ^]^ The optical properties of such structures crucially depend on packing fraction and the structure factor of the assembly.^[^ ^36^, ^37^, ^38^ ^]^ All of these examples rely on the drying of thin liquid films and the studied properties are strongly correlated to the homogeneity of the resulting microstructure.

To date, the microstructure and mechanical properties of microgel‐laden interfaces have been widely studied using Langmuir trough setups, which offer an excellent experimental platform with a flat fluid interface and controlled conditions. These setups involve fluid interface‐assisted assembly by means of lateral compression, often followed by deposition onto solid substrates (Langmuir‐Blodgett deposition). While studies on microgels with various sizes, charge, and crosslinker densities (softness) have yielded valuable insights into the behavior of soft colloids at different fluid interfaces,^[^ ^39^, ^40^, ^41^ ^]^ very little is known about how these films dry,^[^ ^37^ ^]^ even though drying effects could significantly alter the microstructure of a microgel‐laden fluid interface upon transfer onto solid substrates.^[^ ^42^, ^43^ ^]^ In particular, to our knowledge, there has not yet been a study that examines in situ drying dynamics at the single‐microgel level. The research gap exists primarily because visualizing interfaces deformed by soft micro‐units is difficult, and a comprehensive theoretical framework that describes the complex physics during drying is yet to be established.^[^ ^44^, ^45^, ^46^, ^47^ ^]^

In this work, we use a combination of video microscopy and thin film interference to study the drying process of microgel interfacial films on differently modified substrates (hydrophobic vs hydrophilic). By considering micron‐sized silica‐PNIPAM CS microgels^[^ ^48^ ^]^ of comparable sizes and different crosslinker densities (i.e., softness), we trace the drying dynamics at the single‐microgel level and elucidate the interplay between two key elements: the microgel‐to‐substrate adhesion and the capillary forces experienced by microgels in an immersed state (immersion capillary force). Relatively homogeneous drying is reported for films containing microgels with a low crosslinker density drying on hydrophobic substrates, whereas significant alterations of the original microstructures occur when highly crosslinked microgels are deposited on hydrophilic solid surfaces. Similar effects during the deposition of other soft films (e.g., lipid‐laden interfaces) have been observed.^[^ ^49^ ^]^ Understanding the behavior of such soft colloids in thin films not only enhances fundamental knowledge but also has practical implications across various industries, such as microfluidics, surface nanopatterning, biomedical devices, food, cosmetics, and coating.

## 2 Results and Discussion

### 2.1 Experimental Design

Predicting how soft interfacial films dry onto solid substrates is far from trivial. In our model system consisting of microgel‐laden interfaces transferred onto solid substrates, a rich drying scenario emerged depending on 1) the surface chemistry of the substrate, 2) the softness of the individual microgels, and 3) the surface pressure of the microgel‐laden interface. We adjusted these parameters by 1) functionalizing the solid surface, 2) tuning the amount of crosslinker during microgel synthesis and 3) compressing the colloidal films at the fluid interface.

**Figure 1:**
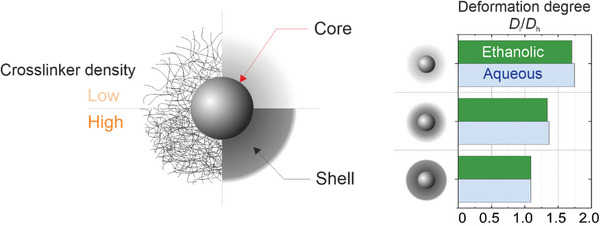
CS microgels with shells of different softness. Left: Schematic illustration of the structure of CS microgels with different crosslinker densities. The solid core (silica, illustrated by the grey sphere) is surrounded by crosslinked PNIPAM shells that are simplified by grey coronas. Dark and light coronas denote high and low crosslinking density, respectively. Right: Degree of lateral deformation at the air/water interface (D_i_/D_h_). The microgels were adsorbed to interfaces from ethanolic and aqueous dispersion, with a 30‐min equilibration. The crosslinker density of the PNIPAM shells increases from top to bottom with the sketches representing the three different CS microgels (CS‐low, CS‐medium, and CS‐high).

To achieve high optical contrast we synthesized three batches of CS microgels that are composed of monodisperse, rigid cores, and PNIPAM hydrogel shells of different crosslinker densities: CS‐low (1.0 mol.% crosslinker by design), CS‐medium (2.5 mol.%), and CS‐high (7.5 mol.%). The structure of these microgels is schematically illustrated in **Figure** **1**. These three model microgels share the same batch of cores (silica, diameter *D*_c_ = 437 nm) and have a similar overall hydrodynamic diameter, *D*_h_, of ≈1 µm (measured by dynamic light scattering at 20 °C). For comparison, “classical,” purely organic microgels without solid cores were also synthesized and investigated. More details about the preparation and characterization can be found in the Experimental Section. The variation in crosslinker density of PNIPAM‐based microgels is clearly observed in the temperature‐dependent hydrodynamic diameter and compression isotherms, as previously reported by several studies.^[^ ^41^, ^50^, ^51^, ^52^, ^53^ ^]^ This difference in crosslinker density among the microgels also becomes apparent through the degree of lateral deformation at the air/water interface, driven by interfacial tension. In this study, we use the term “softness” to describe the deformation behavior of model microgels across diverse scenarios, with CS‐low being the softest of all and CS‐high the stiffest. However, it is important to note that strictly, at interfaces, we deal with a non‐isotropic softness within the polymeric network.^[^ ^54^ ^]^ Additionally, the crosslinker density is not homogeneous within the shell, being higher near the core and lower in the outer layer. Despite these complexities, the primary determinant of microgel “softness” is known to be the crosslinker density.^[^ ^52^ ^]^

When CS microgels adsorb at the air/water interface, they tend to cluster, likely due to their irregular meniscus. This lateral attraction, commonly observed in colloids at fluid interfaces as a result of meniscus undulations, can be described using a multipole expansion. The leading quadrupolar term in this expansion produces a net attractive force.^[^ ^9^, ^10^, ^11^ ^]^ The depth of the potential well (where the capillary attraction overcomes the electrostatic repulsion), is calculated to be 10^4^ k_B_T for a solid particle with 1 µm diameter and 50 nm deviations in the meniscus,^[^ ^55^ ^]^ where k_B_ is the Boltzmann constant. However, this attraction is reported to be reduced for porous materials (such as microgels) by an order of magnitude.^[^ ^7^ ^]^ The degree of lateral deformation in dependence of softness is quantified by the ratio between the interfacial diameter at the air/water interface, *D*_i_, normalized by *D*_h_. We define *D*_i_ as the mean nearest center‐to‐center distance, *D*_c‐c_, at surface pressure Π ≈ 0 mN m^−1^, under the condition that the microgels are clustered, i.e., already in contact.

The values of *D*_i_/*D*_h_ are shown in Figure 1 and Table S1 (Supporting Information). The *D*_i_ measurements were done by reflected light microscopy with 30 min of equilibration time after the adsorption of CS microgels at the air/water interface from ethanolic dispersion (ethanol as the spreading agent) as well as from aqueous dispersion (spontaneous adsorption from subphase), where they form clusters, a precondition for our definition of *D*_i_. These clusters of CS microgels and their corresponding compression isotherms are shown in Figure S1 (Supporting Information). While the CS‐low microgels assume ≈1.7 times their (lateral) diameter at the fluid interface as compared to *D*_h_, the stiffest microgels, CS‐high, show only little deformation at the interface (*D*_i_/*D*_h_ ≈ 1). The use of ethanol as spreading agent as compared to spontaneous adsorption from the purely aqueous subphase has very little effect on the degree of deformation at the interface. The degree of deformation for CS‐low (1 mol.% BIS) is similar to values found for significantly higher crosslinked CS microgels (5 mol.% BIS) in the literature.^[^ ^50^ ^]^ This difference can be attributed to the much larger dimensions of the CS microgels in the present work. We assume that the contact radius *a* scales approximately with *R* ^2/3^ which is the scaling known from the contact deformation model by Johnson, Kendall, and Roberts (JKR‐model).

Using ethanol as a spreading agent,^[^ ^56^ ^]^ the CS microgels were adsorbed at the air/water interface of a Langmuir–Blodgett trough (Microtrough G2, Kibron Inc.), and a surface‐modified solid substrate was immersed (90° to the interface and parallel to the barriers) using a dipper. For the preparation of hydrophilic substrates, standard microscopy glass slides were thoroughly cleaned and subsequently plasma‐treated right before the experiment. For the hydrophobic substrates, the glass slides were RCA‐cleaned^[^ ^48^ ^]^ and fluorinated via chemical vapor deposition. Details regarding the microgel synthesis, Langmuir‐Blodgett deposition, and substrate preparation are in the Experimental Section. The microgel‐laden interface was later compressed uniaxially by means of lateral barriers to reach a given Π. Three values of Π are considered to achieve various *D*_c‐c_ (i.e., compression state): ≈10, 20, and 30 mN m^−1^, to which we will refer as “low,” “mid,” and “high Π.” The corresponding compression isotherms for all three CS microgel systems can be found in Figure S1 (Supporting Information). Video microscopy of the drying interface was performed by transferring the microgel‐laden interface rapidly (dipper speed: 279 mm min^−1^) onto the solid substrate at constant surface pressure.^[^ ^53^, ^57^, ^58^ ^]^ Images of the drying microgel‐laden interfaces (frequently known as microgel monolayer or microgel films) were taken in situ immediately after the transfer using reflected brightfield microscopy. Even at a high Π, i.e., high packing fractions, individual CS microgels remain well‐resolved due to the thick PNIPAM shells and the silica cores providing large center‐to‐center distances and high optical contrast. Under our imaging conditions, the drying microgel films also exhibited iridescent colors resulting from thin film interference.^[^ ^22^ ^]^ These colors can be used to map the local height of the microgel‐laden interface with respect to the underlying substrate (details about this method are given in the Experimental Section).

### 2.2 Effect of Hydrophilicity/Hydrophobicity of the Substrate

Using a combination of 3D superresolution fluorescence microscopy and computer simulations, Hoppe Alvarez et al.^[^ ^59^, ^60^ ^]^ and Shaulli et al.^[^ ^61^ ^]^ have demonstrated that thermoresponsive microgels maintain their native spherical shapes on hydrophilic substrates (against an aqueous phase). Conversely, on hydrophobic substrates, which have lower surface energy and prefer to avoid contact with water, the same microgels undergo significant deformation, creating larger contact areas between the microgels and the substrate. This is attributed to the fact that the contact of a hydrophobic surface with amphiphilic polymer chains is energetically more favorable than with water molecules. Recent simulations of tethered homopolymer chains in planar brushes and block copolymer micelles also revealed energetically favorable substitution of surface‐water to surface‐polymer contacts on hydrophobic surfaces, promoting polymer adsorption.^[^ ^62^ ^]^

**Figure 2:**
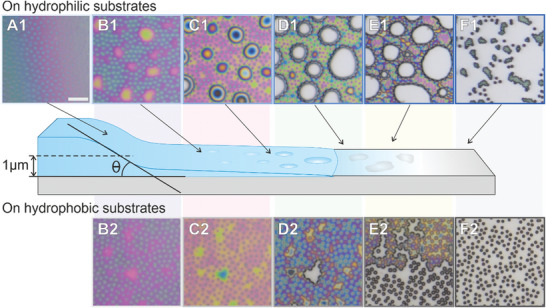
Sketch illustrating the drying of microgel films (middle row) and reflected light microscopy images of CS‐medium films at mid‐Π (≈20 mN m^−1^) transferred onto hydrophilic (A1–F1) and hydrophobic substrates (B2–F2). The colors are due to thin film interference and will be later used to determine the local height. The scale bar corresponds to 5 µm. All microscopy images show the same magnification. θ is the receding wetting angle of the meniscus. Equal letters refer to similar film thicknesses deduced from similar colors.

**Figure** **2** depicts the drying of a film containing CS‐medium at mid‐Π on hydrophilic (Figure 2, panels A1‐F1) and hydrophobic (Figure 2, panels A2‐F2) substrates at various stages of the drying process (see Videos S1 and S2, Supporting Information). Confocal scans of the meniscus cross‐section are used to measure the slope of the meniscus during drying (see Experimental Section), i.e., the receding wetting angles θ_1_ < 1° (hydrophilic substrate, Figure S2, Supporting Information) and θ_2_ ≈ 2° (hydrophobic substrate, Figure S3, Supporting Information). Panel A1 of Figure 2 shows the microstructure of the microgel film on the inclined interface, which is indicated by the color gradient. Following this stage, we report the formation of a thin fluid layer where the microgel‐laden interface aligns parallel to the solid surfaces, evidenced by the positioning of all microgels in the focal plane (panels B1 and B2). As the level of water lowers further (panels B–D), the color of the core region (i.e., silica core plus swollen microgel around it) becomes increasingly distinct from the rest, signifying different surface elevations. On hydrophilic substrates, regions with low microgel concentration (empty areas) grow in size as the drying proceeds (hereinafter called hole formation, see Figure 2, panels C1–E1), pushing the microgels against each other until the film completely dries (Figure 2, panel F1). This hole formation appears to be largely influenced by fluid dynamics, as evidenced by the circular shape of the enlarging holes, which are also observed for both micron‐sized hard sphere‐like systems^[^ ^10^ ^]^ and microgel systems.^[^ ^63^ ^]^ Remarkably, the migration (XY displacement) of the microgels is negligible when the drying takes place on hydrophobic substrates (Figure 2, panels C2–F2). The characterization is repeated at different values of Π. The corresponding microscopy images are depicted in Figure S4 (Supporting Information). We quantify the effect of water evaporation on the microgel films by measuring *D*_c‐c_ before and after the drying (Figure S5, Supporting Information). *D*_c‐c_ changes significantly when the underlying substrate is hydrophilic indicating higher mobility as compared to the hydrophobic case. This is in general agreement with the findings of Hoppe Alvarez et al.^[^ ^59^, ^60^ ^]^

**Figure 3:**
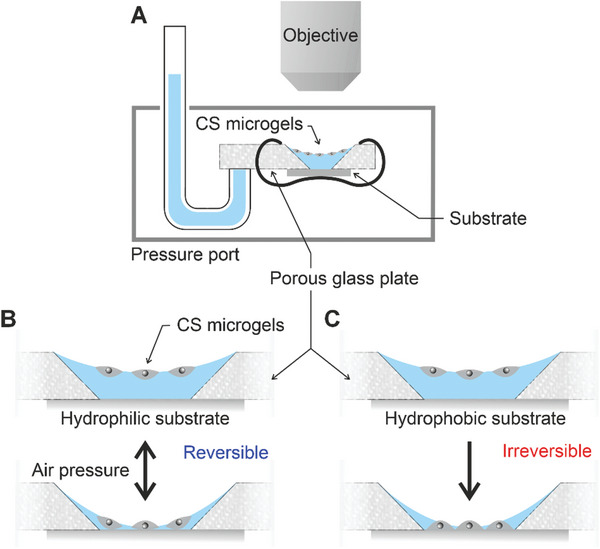
Schematic illustrations of a modified thin film pressure balance (TFPB) setup (A) and the microgel‐laden interface oscillation experiment by pressure modulation using a hydrophilic substrate: far away from the solid substrate (B) and in the thin film on the substrate (C). Videos recorded for experiments on a film of CS‐medium are provided in the Supporting Information.

To better understand and control the drying and rewetting process, we mimicked the contact scenarios between the microgel‐laden interfaces and solid substrates using a modified thin film pressure balance (TFPB) equipped with a porous glass plate and connected to a capillary tube filled with water (**Figure** **3**A; Figure S6, Supporting Information) similar to the one used by Ciunel et al.^[^ ^64^ ^]^ and Schelero et al.^[^ ^65^ ^]^ The glass plate was soaked with water and had a truncated hole, where the air/water interface was created on the top and the substrate was placed on the bottom. In analogy to the CLSM measurements, the microgel dispersion was spread at the air/water interface. The porous glass plate was placed in a pressure cell and by varying the air pressure, the setup enabled the on‐demand position control of the microgel‐laden interface, which extended from a few mm above the solid substrate down to thin wetting films comparable to the microgel size. In the presence of a hydrophilic substrate (Figure 3B), i.e., weak microgel‐to‐substrate adhesion, microgels trapped in the thin fluid film could be released back to the air/water interface on demand by refilling the wetting film with water via a decrease of the pressure in the cell. This phenomenon was most clearly observed in the Videos S1 and S2 (Supporting Information), where the mobility of the microgel‐laden interface is evident as it floats back up. Conversely, the TFPB experiment conducted on hydrophobic substrates (Figure 3C) revealed that the microgel monolayer was trapped immediately due to the strong adhesion. Refilling the wetting film with water did not lead to desorption and floating back of the microgels to the air/water surface was not possible. Instead, when attempted (repeatedly increasing and decreasing the position of the microgel‐laden interface), it resulted in multilayers of the microgels, see Videos S1 and S2 (Supporting Information) and Figure S7 (Supporting Information). This spontaneous and favored adsorption of microgels from the water phase onto a hydrophobic substrate was also reported in the drying experiment on microgel‐laden droplets.^[^ ^66^ ^]^

**Figure 4:**
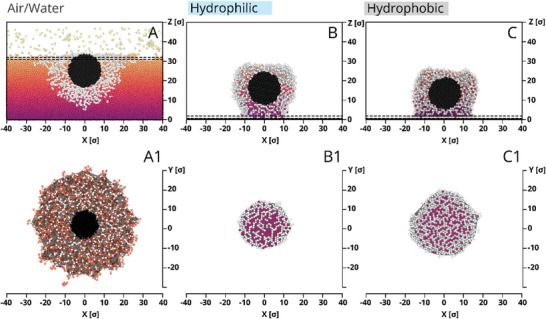
Snapshots of a selected CS microgel in the monolayer obtained at low Π before (A,A1) and after drying on hydrophilic (B,B1) and hydrophobic (C,C1) substrates. The upper row demonstrates a side view (cross‐section through the center of mass). The bottom row depicts thin layers of the microgels shown by dashed lines in the upper row, i.e., they are upper view (A1) and contact area on hydrophilic (B1) and hydrophobic (C1) substrates. The purple dots in (B,B1) and (C,C1) correspond to residual water.

Using molecular dynamics simulations with a coarse‐grained model and explicit solvent, we further verified the drying scenarios of microgel‐laden interfaces (details are presented in Supporting Information). Specifically, we constructed a system based on the experimental results in which a monolayer of 16 CS microgels was created (Figure S8, Supporting Information). **Figure** **4** illustrates the microgel deformation at the air/water interface before water evaporation (panels A,A1) and its shape on hydrophilic (panels B,B1) and hydrophobic (panels C,C1) substrates after water evaporation. The term “water” in our description refers to a simplified model, specifically a Lennard–Jones truncated and shifted (LJTS) fluid. Figure 4 presents two significant aspects: 1) the lateral size of microgels decreases upon drying, and 2) the contact area of dry microgels on hydrophobic substrates is larger compared to hydrophilic substrates. The enhanced contact area on hydrophobic substrates persists at all stages of the microgel adsorption, from the initial solvent‐rich stage to after drying, resulting in stronger molecular “friction” between the microgel and substrate. These findings support the experimentally observed structural quenching (maze‐like cracks) upon solvent evaporation and the reduced mobility of microgels on hydrophobic substrates.

**Figure 5:**
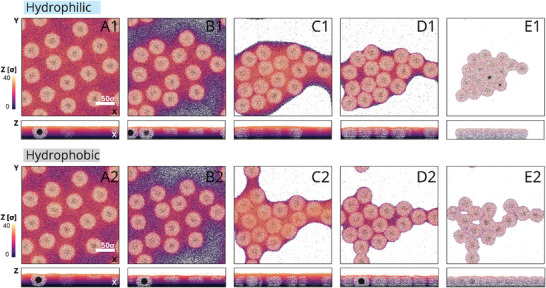
Illustration (top views) of the different stages of microgel film obtained at low Π drying on hydrophilic (A1–E1) and hydrophobic (A2–E2) substrates as revealed by computer simulations. The narrow panels below the top views are the corresponding side views, i.e., cross‐sections of each panel along Z. The height of the film with residual water is depicted by the vertical color bar. White regions in the snapshots correspond to the bare substrate after water evaporation.

Analogous to Figure 2, the simulation‐assisted drying behaviors of the microgels on differently modified substrates (hydrophobic vs hydrophilic) during a continuous water evaporation process are illustrated in **Figure** **5** (panels A–E). It is important to note that the evaporation of water from the interface is not uniform, with the most rapid drying occurring in the regions between the microgels, where a lower polymer concentration enables efficient evaporation. This asymmetry results in the formation of a drying front (hole formation), prompting XY displacement of microgels. The hole formation could be further influenced by disjoining pressure, which stabilizes the thin water film on the substrate. On hydrophilic substrates, the disjoining pressure is positive, which stabilizes the film and prevents local film instabilities. However, for hydrophobic substrates, the disjoining pressure should become negative, as previously reported,^[^ ^67^ ^]^ resulting in an unstable film. In other words, at a critical thickness of the film (generally thicker on the hydrophobic substrate), the evaporation of water triggers the instability leading to the de‐wetting of the water film.

The simulation results show that the mobility of the microgels on the hydrophobic substrate is reduced (Figure 5, panels A2–E2). Here, we also see that the microgel cluster in a dry state (Figure 5, panel E2) is less compact compared to the case on the hydrophilic substrate (panel E1). The compactization, which is driven by the minimization of the surface energy of the drying microgels, proceeds faster on the hydrophilic substrate due to the higher mobility of the microgels. The discrepancy in microgel behavior on various substrates is quantified by the parameter *D*_c‐c_ in Table S4 (Supporting Information). However, it is evident that the microgel‐to‐substrate adhesion is insufficient to prevent XY displacement in both cases. This might be because water evaporation in the experiments leads to a far out‐of‐equilibrium quenched (or “frozen‐in”) state of the system, whereas in the simulations, interaction parameters used allow equilibration of the system even in a dry state. See Model and Simulation Details in Supporting Information for more details (Figures S9–S14, Supporting Information).

### 2.3 Effect of Microgel Softness

**Figure 6:**
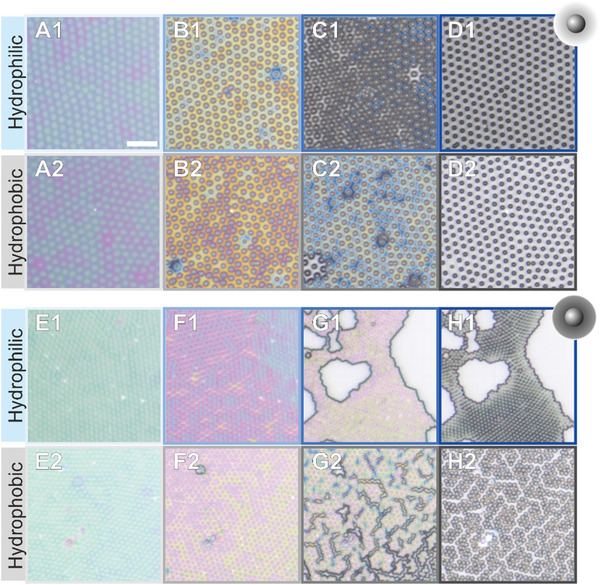
Reflected light microscopy images of CS‐low (A–D) and CS‐high microgel films (E–H) at mid‐Π (≈20 mN m^−1^) transferred onto hydrophilic (A1–D1, E1–H1) and hydrophobic substrates (A2–D2, E2–H2) during the drying process. The scale bar corresponds to 5 µm.

We take a further step to characterize the drying of soft films and compare two CS microgels with different crosslinker densities, i.e., softness. **Figure** **6** illustrates different stages of the drying process of CS‐low and CS‐high microgel films at mid‐Π. For “softer” and thus more deformable microgels (CS‐low, Figure 6, panels A–D), the surface chemistry of the substrate has minimal‐to‐no influence on the spatial rearrangement upon drying. Significant microstructural changes, as reported for CS‐medium films (A1–F1 of Figure 2), occur for CS‐low only at high Π on hydrophilic surfaces (Figure S15, Supporting Information). Conversely, microgels with “harder,” less deformable shells (CS‐high, Figure 6E–H) undergo significant XY displacements as they dry, irrespective of the hydrophobicity of the substrate. In particular, an abrupt and sudden collapse of microgels (i.e., a fast and large hole formation) is observed when the film dries onto hydrophilic substrates and the thin layer of fluid reaches a certain thickness. On the other hand, on hydrophobic substrates, the microgels tend to lean and/or slide toward one another, creating maze‐like cracks. In analogy to Figures S4 and S5 (Supporting Information), we also report in Figures S15–S17 (Supporting Information) the microscopic images and *D*_c‐c_ for different Π values. (In the presence of clusters *D*_c‐c_ refers to the distance between the microgels in the clusters.)

To elucidate whether the observed effects rely on the core‐shell morphology of our microgels, which affects at least the total deformability of the microgels, we also studied purely polymeric microgels of similar and smaller total sizes. One would anticipate that the latter microgels would deform more easily and thus exhibit lower mobility during drying. However, similar changes in microstructure as for our CS microgels during drying were observed for pure PNIPAM microgels of comparable sizes (Figures S18 and S19, Supporting Information) as well as smaller CS microgels (Figure S20, Supporting Information) in the mid‐high Π regime.

### 2.4 Interplay Between the Adhesion and Immersion Capillary Force

#### 2.4.1 Qualitative Picture

**Figure 7:**
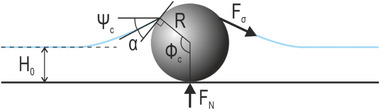
Scheme of forces acting on a small particle with a radius of R, immersed in a thin water film with a height of H_0_. The angles between the interfaces can be characterized by the particle contact angle, α, and the wetting angle ϕ_c._ The particle is under the influence of forces that arise from the surface tension, F_σ_, of the liquid along the wetted perimeter and the upward normal force exerted from the solid substrate, F_N_.

When a solid particle is immersed in the liquid film, it is under the influence of many forces, such as gravity, and forces that come from the presence of the liquid around the particle, such as buoyancy and surface tension. For small particles, e.g., diameter below 1 µm (which is the case we are investigating), the influences of gravity and buoyancy become negligible. Thus, only the force arising from the surface tension, *F*_σ_, of the liquid along the wetted perimeter (and the upward normal force exerted from the solid substrate, *F*_N_, becomes relevant, where their force balance is given by:^[^ ^68^ ^]^

(2) $$F_{N\;}=2\pi R\sigma \sin\phi_{c}\sin\left(\phi_{c}+\alpha \right)$$

where *R* is the radius of the particle, σ is the surface tension of the liquid, and all contact angles involved, governed by the wetting properties of the particle surface, namely the meniscus slope angle, $\Psi_{c}$ not to be confused with the receding wetting angle θ from Figure 2, particle contact angle, α, and the wetting angle ϕ_c_ where $\Psi_{c}$ + α +  ϕ_*c*_ =  180°,^[^ ^68^ ^]^ as depicted in **Figure** **7**. In the presence of another particle (same type and size) in the vicinity, the particles are under the influence of the immersion capillary force. The lateral capillary interaction between the two particles can be described as:

(3) $$\Delta W\approx -2\pi \sigma Q^{2}K_{0}\left(qD_{c-c}\right)$$

where the “capillary charge” is *Q* ≡ *R_c_* sin $\Psi_{c}$ and *K*_0_ is the modified Bessel function of the second kind and zeroth order, in addition:

(4)

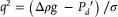

where ∆ρ is the difference between the densities of the two fluids (air and water) and *P*_d_' is the derivative of the disjoining pressure as a function of the height of the film.^[^ ^10^, ^11^ ^]^ The disjoining pressure becomes negligible when *H*_0_ exceeds 100 nm for the wetting film.^[^ ^9^ ^]^ The immersion capillary force exerted on the two particles, which is the derivative of the lateral capillary interaction:^[^ ^10^ ^]^

(5) $$F=-\frac{d\Delta W}{dD_{c-c}}\approx -2\pi \sigma Q^{2}qK_{1}\left(qD_{c-c}\right)$$

where K_1_ is the modified Bessel function of the first order.

For rigid spheres with dimensions relevant to our CS microgels, i.e., *R*_c_ < *R* < *R*_h_, the lateral capillary interaction (−∆*W*) reaches theoretical values that exceed the thermal energy by up to eight orders of magnitude, depending on $\Psi_{c}$ and *D*_c‐c_. Since our CS microgels consist of a solid core surrounded by a soft and deformable microgel network where the contact and meniscus slope angle cannot be easily determined and potentially also depend on the water content in the microgel shell, capillary interactions are expected to significantly differ from that of rigid spheres. In a recent study on the drying of microgel dispersions within capillary tubes a drying behavior in between of colloidal dispersions and polymer solutions has been found.^[^ ^69^ ^]^ Due to the microgel's abilitiy to deform, deswell and interpentrate, their drying behavior from bulk dispersion is dominated by molecular scales. To some extent we expect this to be also relevant for drying of monolayers confined in a thin liquid film.

**Figure 8:**
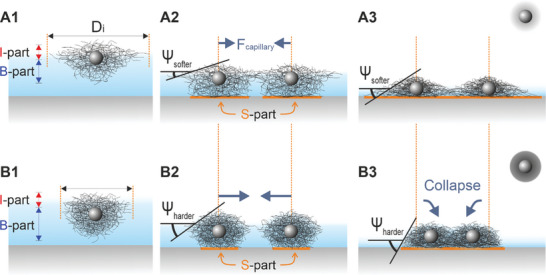
(A1,B1) Schematic illustration of microgels with a low (A1) and high crosslinker density (B1) at the air/water interface and their interfacial diameter, D_i_. (A2,B2) Upon drying, at the same D_c‐c_ and a given film height (D_h_ > H > D_c_), softer microgels (A2) have a larger contact area to the substrate (highlighted in orange) and lower meniscus slope angle (Ψ) compared to harder microgels (B2). (A3,B3) Soft microgels are, therefore, more likely to stay in position (A3) than to collapse (B3).

To elucidate the physical phenomena that occur during the drying of our microgel films, we first illustrate how we picture the microgel structure and deformation at air/water interfaces for “softer” and “stiffer” CS microgels, as depicted in **Figure** **8** panels A1 and B1, respectively. Our understanding described here will be quantified and further validated in the following section. For simplicity, the microgels can be considered as made of three parts: I‐part (which interacts with the air/water interface), B‐part (which interacts with the bulk water phase), and S‐part (which interacts with the solid surface). Due to the lateral deformation (see, e.g., Figure 1) caused by interfacial tension, leading to non‐isotropic softness within the polymeric network, the portion at the interface (I‐part, Figure 8 panels A1, B1) experiences more stretching than the portion in bulk (B‐part).^[^ ^54^ ^]^

As water evaporates from the thin fluid layer (see, e.g., Figure 2, panels C,D), the bottom B‐part of the shells comes in contact with the underlying substrate (S‐part, Figure 8 panels A2, B2). If the resulting adhesion (dominated by the amount of material interacting with the substrate) is not strong enough to counteract the film instability or the de‐wetting of water, microgels migrate (hole formation) and aggregate (see, e.g., Figure 2, panels D1,H1). If the adhesion is strong enough, microgels stay in position in the thin aqueous film, where the local meniscus slope angle, Ψ, and microgel‐to‐substrate contact area (colored orange) increases while the average film thickness decreases as the drying proceeds, increasing the immersion capillary force exerted among microgels. At the same *D*_c‐c_ and film height (*D*_h_ > *H* > *D*_c_), the harder microgels will be under a stronger immersion capillary force due to the steeper meniscus slope angle (Ψ_harder_ > Ψ_softer_) and have weaker microgel‐to‐substrate adhesion (i.e., smaller contact area) due to the high elastic modulus of the polymer network. At a certain height, the immersion capillary force (long‐ranged) and disjoining pressure (short‐range) of the film can surpass the microgel‐to‐substrate adhesion, causing the film to rupture or de‐wet, i.e., microgels to migrate (distance traveled > *D*_c‐c_) or collapse onto one another (distance traveled < *D*_c‐c_, Figure 8 panel B3), i.e., there is a gradual decrease in *D*_c‐c_ as the effective volume of microgel reduces due to evaporating water. If the adhesion persists stronger than the acting immersion capillary force, the migration of microgels does not occur (Figure 8 panel A3, no XY displacement) and the microstructure of the microgel‐laden interface is better preserved. Therefore, if the goal is to produce dried replicas of the interfacial microstructure, soft microgels, and hydrophobic (or oppositely charged) substrates are to be used rather than harder microgels and hydrophilic substrates under fast drying conditions.

#### 2.4.2 Quantitative Validation: Thin Film Interference and Particle Tracking

**Figure 9:**
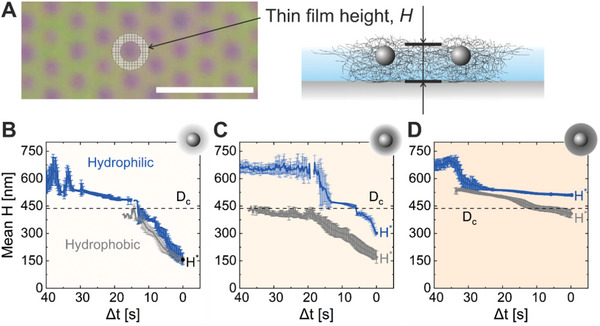
Film height, H, shown in an exemplary frame (A, left) and in a sketch (A, right). The scale bar corresponds to 5 µm. Evolution of the height profiles of ten randomly chosen CS microgels in corresponding films drying on the hydrophilic (blue) and hydrophobic (grey) substrates for CS‐low (B), CS‐medium (C), and CS‐high (D). Δt represents the time difference, with 0 as the reference point, where H^*^ is observed.

For a more quantitative validation, we measure film height, *H*, during the drying of the microgels after the formation of a thin fluid layer. We consider interfaces filled with CS microgels of different softness (CS‐low, CS‐medium, and CS‐high) at a comparable *D*_c‐c_ (≈ 840 nm, at the air/water interface), and extract the instantaneous velocity *V* of the particles (i.e., the displacement of the core's center between consecutive frames) and the height of the microgel film as it dries. The height profiles of the drying films were traced by their apparent colors, which stem from thin film interference,^[^ ^22^, ^70^ ^]^ under the assumption that the interface is perfectly flat, and the refractive index of the thin film equals that of water throughout the drying process. However, it is important to note that as the drying progresses, the height profiles will be increasingly underestimated due to the increasing effective refractive index of the film (increasing polymer volume fraction). The high refractive index and large size of the silica cores introduce further complexity. To simplify the calculation, we masked the core areas (arbitrarily determined, ≈600 nm in diameter) and only considered the height of the shell area (*H* in **Figure** **9**A) for radial averaging. We define *H = H* ^*^ (critical height), when the microgels start to move from their original XY position. The methodology is described in detail in the Experimental Section.

Figure 9 (panels B–D) shows the evolution of the height profiles of microgel‐laden interfaces during drying on hydrophilic (blue) and hydrophobic (grey) substrates as a function of a normalized time, where *t* = 0 s is the onset of XY displacement of the individual microgels. The corresponding instantaneous velocities are in Figure S21 (Supporting Information). All height profiles exhibit plateau regions near *H* = *D*_c_, possibly due to the combination of 1) the elasticity of the microgel networks supported by the core, 2) increased polymer volume fraction, 3) artifact of radial averaging (due to the changes in Ψ as the drying proceeds). Notably, the height of the microgel films is generally lower on hydrophobic substrates compared to hydrophilic substrates because of the stronger microgel‐to‐substrate adhesion and more pronounced deformations of the polymer networks in contact with the solid surface. The critical height *H* ^*^ is also consistently higher for interfaces made of microgels with “harder” shells, as illustrated in Figure 9 (panels B–D) and Table S1 (Supporting Information). The “harder” polymer network appears to resist the thinning of the film, which could also result in a relatively smaller contact area with the substrate and, consequently, weaker microgel‐to‐substrate adhesion and higher mobility during drying. The difference in softness among CS microgels is also evident in the lateral deformation rate at the air/water interface in Figure 1 and Figure S1 and Table S1 (Supporting Information). Our results indicate that the drying of a microgel film involves an intricate interplay among various factors, such as microgel‐to‐substrate adhesion, microgel compression degree, immersion capillary forces, and others, e.g., free energy associated with interface formation, Marangoni flow, drying conditions.^[^ ^71^, ^72^ ^]^ Further quantification of the phenomena requires values of the slope angle of the meniscus and microgel adhesion both of which will change as the drying processes.

## 3 Conclusion

Interparticle interactions among soft micro‐units and their phase behavior at interfaces are of great importance in fundamental interface science, biophysics, and additive manufacturing. Studying such phenomena at fluid interfaces, however, is challenging due to the difficulty in visualizing both the building blocks and the deformed interface, particularly in condensed (or highly compressed) states. Our results provide direct imaging and quantification at the single‐particle level of drying model soft interfaces consisting of microgel monolayers on various solid substrates. Using various crosslinked CS microgels, we demonstrated that the resulting dried microstructure of the microgel monolayers is significantly influenced by the softness of the microgels and the wettability of the substrates. In general, microgels with higher softness better maintain the original assembled structure due to more pronounced deformation at the interfaces. This results in lower capillary forces due to a less curved air/water interface. In addition, increased hydrophobicity of the substrate supports the conservation of the arrangement of the microgels during drying due to enhanced adhesion to the substrate and, consequently, lower mobility during the drying process.

We experimentally showed that the drying dynamics of such monolayers involve an intricate interplay among various factors, including microgel‐to‐substrate adhesion, immersion capillary forces, and the free energy associated with the interface formation. Our results are supported by molecular dynamics simulations, where the hole formation and the varying degrees of deformation, causing differences in their drying dynamics on differently surface‐modified substrates upon solvent evaporation, are visualized. This implies that the interpretation of the “2D” assembly of soft colloids at interfaces must consider the often‐overlooked 3D aspects: how microgels deform in 3D initially at interfaces, undergo further deformation under compression, and continue to deform during the drying process. This perspective has also been emphasized in a recent study on emulsion stabilization using microgels.^[^ ^73^ ^]^ While the presence of the rigid, non‐deformable core increases the operative immersion capillary force and may influence the shell's effective softness, when trapped in thin liquid films (i.e., during drying on a substrate), the deformation of the interface is inevitable, even for submicron‐sized purely polymeric microgels without rigid cores. The system will then react on the microgels to minimize the surface energy configuration.^[^ ^74^ ^]^ Therefore, ex situ results, i.e., data obtained after transfer onto a substrate, should be approached with caution.

To date, there is no generally accepted model for the interactions among soft colloids at interfaces bridging from dilute to condensed (compressed) state. In future investigations, we will explore the interparticle interactions of these soft colloids, especially at the single‐colloid level, using techniques such as optical tweezing. This approach has the potential to enhance our understanding of the complex phase and rheological behaviors exhibited by soft colloids. Furthermore, for applications where soft materials come in contact with more rigid surfaces and undergo dehydration and rehydration (e.g., tissue and food engineering, biomedical application),^[^ ^75^, ^76^, ^77^, ^78^ ^]^ it could be interesting to explore the influence of varying surface roughness or porosity and measure the effective lateral attraction force among differently engineered microgels and their microgel‐to‐substrate adhesion forces in various environment.

## 4 Experimental Section

### Materials

Ethanol (Sigma‐Aldrich, 99.8%), ethanol (Heinrich‐Heine‐University, chemical store, p.a.), tetraethyl orthosilicate (Sigma‐Aldrich, 98%), ammonium hydroxide solution (NH_3_ (aq.), PanReac AppliChem, 30%), ammonium hydroxide solution (NH_3_ (aq.), VWR, 25%), ammonium hydroxide solution (NH_3_ (aq.), PanReac AppliChem, 30%), hydrogen peroxide solution (H_2_O_2_, Fisher Chemical, 30 wt %), rhodamine B isothiocyanate (Sigma‐Aldrich, mixed isomers), methacryloxyethyl thiocarbamoyl rhodamine B (MRB, Polysciences, Inc.), (3‐aminopropyl)trimethoxysilane (Sigma‐Aldrich, 97%), 3‐(trimethoxysilyl)propyl methacrylate (MPS, Sigma‐Aldrich, 98%), 1H,1H,2H,2H‐perfluoroctyltriethoxysilan (PFOES, J&K Scientific, 97%), *N*,*N*’‐methylenebisacrylamide (BIS, Sigma‐Aldrich, 98%), and potassium peroxodisulfate (KPS, Sigma‐Aldrich, 99%) were used as received. Water was purified by a Milli‐Q system (18.2 MΩ cm) and *N*‐isopropylacrylamide (NIPAM, TCI, 97%) by recrystallization from cyclohexane (Fisher Scientific, 99.8%).

### Synthesis

The synthesis of CS microgels was done via seeded precipitation polymerization. In brief, silica particles were synthesized using the well‐known Stöber procedure and their surfaces were modified with MPS. RITC dye was incorporated into the particles. The diameter, *D*_c_, measured by transmission electron microscopy (TEM) was 437 ± 20 nm. The PNIPAM shell encapsulation of the cores proceeded through seeded precipitation polymerization at 70 °C with various feed concentrations of BIS relative to NIPAM. Specifically, 1.0, 2.5, and 7.5 mol.% BIS was used for the synthesis of CS‐low, CS‐medium, and CS‐high, respectively. The exact amounts of chemicals used are listed in **Table** **1** and the synthesis protocols for the micron‐sized CS microgels are detailed elsewhere.^[^ ^48^ ^]^ After purification via repeated centrifugations and re‐dispersion cycles, the dispersion was freeze‐dried, re‐dispersed in ethanol at a concentration of 5 w/v.%, and left overnight on a 3D shaker before use.

**Table 1:** Respective amounts of chemicals used for the synthesis of CS‐low, CS‐medium, and CS‐high.

	Core		Core‐shell				
Sample name	Particle/ethanol [g mL^−1^]	Added [mL]	H_2_O [mL]	NIPAM [g]	BIS [g]	KPS [g]	*D*_h_@20 °C [nm]
CS‐low	0.53	1	255	1.0014	0.0135	0.0256	1114.1
CS‐medium				1.0012	0.0343	0.0255	1003
CS‐high				1.0004	0.1022	0.0258	1053.2

The pure PNIPAM microgels were synthesized via precipitation polymerization according to previously published work.^[^ ^19^, ^20^ ^]^ The synthesis protocols were modified for dye incorporation for smaller microgels (*s*MG, *D*_h_ ≈ 800 nm) (Figure S4, Supporting Information) as described in^[^ ^42^ ^]^ as well as for larger microgels (*l*MG, *D*_h_ ≈ 1500 nm) (Figure S5, Supporting Information). *l*MG was synthesized with 2.11 g of PNIPAM and 60 mg of BIS (2 mol.%), dissolved in 125 mL of water. The mixture was injected through a 0.2 µm Nylon syringe filter into a three‐neck round‐bottom flask equipped with a reflux condenser and a magnetic stirrer. 250 µL of MRB dye aq. solution (1 mg mL^−1^) was added to the flask. The mixture was heated to 45 °C and equilibrated for an hour while purged with nitrogen under stirring. 0.1054 g KPS in 5 mL water was added to the flask through a 0.2 µm Nylon syringe filter. After initiation, the temperature was ramped up to 65 °C in 40 min. The reaction was kept overnight under stirring at 65 °C. The dispersion was then filtered through glass wool. The synthesized PNIPAM microgels were dialyzed against water for 2 weeks, freeze‐dried, and re‐dispersed in ethanol (1 w/v.%).

### Methods—Dynamic Light Scattering (DLS)

The hydrodynamic diameter, *D*_h_, of the CS microgels was determined using a Zetasizer Nano S (Malvern Panalytical). The device was equipped with a HeNe laser (4 mW, 633 nm) and a temperature‐controlled jacket for the sample. Measurements were performed at a scattering angle of 173° at 20 °C. Three measurements were performed. Values of *D*_h_ reported were averaged from the z‐averages obtained from the measurement software.

### Methods—Glass Substrates for Monolayer Transfer

For the preparation of glass substrates with a hydrophilic surface, standard microscopy glass slides were thoroughly cleaned and rinsed using water and ethanol, and then plasma‐treated prior to the monolayer transfer. For the hydrophobic surface modification, the glass substrates were RCA‐cleaned^[^ ^79^, ^80^ ^]^ and surface‐modified via chemical vapor deposition. 200 µL of PFOES was stored with the cleaned glass substrates in a desiccator overnight under vacuum (25–30 mbar). The glass substrates were then placed in an oven at 120 °C for an hour to ensure covalent bonding. Afterward, the substrates were washed in ethanol in an ultrasonic bath to remove unreacted silane molecules. The contact angle with 5 µL water droplet was measured by a drop shape analyzer (DSA 25, Krüss) at room temperature (25.5–26.6 °C, relative humidity 35–46%). The contact angle of the freshly plasma‐treated glass substrate (within 10 mins) was 3.5 ± 0.8° and that of the PFOES‐modified glass substrate was 103 ± 0.9°.

### Methods—Monolayer Transfer and Optical Microscopy

The transfer of the CS microgel monolayer on the differently modified substrates was carried out using a Langmuir–Blodgett deposition trough (Microtrough G2, Kibron Inc.) equipped with a film balance, two Delrin barriers, a dip coater, and an acrylic cover box. The monolayer deposition was done rapidly maintaining the measured surface pressure (surface pressure changed during the transfer: 1.4 ± 1.1 mN m^−1^, compression speed: 187 mm min^−1^, dipper speed: 279 mm min^−1^) positioned at 90° to the air/water interface and parallel to the barriers. The “wet” monolayer on the glass slide was then placed under a light microscope (Eclipse LV150N, Nikon) equipped with a camera (DS‐Fi3) and a 100× objective for the in situ monitoring of the drying of the monolayer (≈5 frames per second). Pure PNIPAM microgels were investigated under a fluorescence microscope (Olympus IX73) equipped with a mercury lamp, a fluorescence filter set, a CMOS camera, and a 60× objective.

### Methods—Confocal Laser Scanning Microscopy

XZ in situ time series scans of the drying CS microgel monolayers were acquired using a Zeiss inverted LSM880 Airyscan microscope system (Zeiss Microscopy GmbH), equipped with a Plan‐Apochromat 40x/0.95 dry objective lens. Microgel monolayers were prepared as described at the air/water interface in a crystallizing dish and transferred on cover glasses as described in Ref. [48] and were mounted immediately on the microscope motorized stage. The time series scans were started in regions that were not in the dried state. Two simultaneous acquired channels were set up in fluorescence and reflective mode to observe the Rhodamine B labeled silica cores in the monolayer and their distance to the cover glass and the water meniscus, respectively. 561 nm was used at 3% intensity as an excitation laser line with a PMT detector set at a range of 580–670 nm for the acquisition of the fluorescence. For simultaneous acquisition of the laser reflection, another GaAsP detector was set at a range of 540–580 nm overlapping with the 561 nm excitation laser line. The general acquisition parameters were set as follows: The calculated pinhole size was used at 0.44 airy units. The x‐axis pixel size was set to 208 nm at a total scan length of 213 µm. The z‐axis covered a range of 49 µm as 100 slices with an interval of 492 nm. The scans were performed in line scan fast Z mode at a scan speed of 2.05 µsec/pixel resulting in an average framerate of 1.08 s/frame. The receding wetting angles, θ, of the drying microgel thin films (Figures S2 and S3, Supporting Information) were measured using ImageJ (Angle tool, version 1.53k, National Institutes of Health, USA).

### Methods—Thin Film Pressure Balance

To investigate the adsorption and desorption of microgel monolayers at the water/substrate interface, a custom‐built thin film pressure balance (TFPB) using the porous glass plate method^[^ ^81^, ^82^ ^]^ in wetting configuration^[^ ^64^ ^]^ was used at 22 °C. The centerpiece was a porous glass plate (pore size 10–16 µm, porosity P16 (ISO 4793)) with a drilled hole of diameter of ≈1 mm. The porous glass plate was attached to a glass capillary tube (film holder), which was filled with water in a way that the water could flow from the porous glass plate to the capillary tube and vice versa. The substrate of interest (various hydrophilic glasses, silicon wafers, and hydrophobic glass) was placed underneath the hole of the porous glass plate and was fixed with a stainless‐steel clamp, as shown in Figure S6A (Supporting Information). The monolayers of microgels were prepared for the confocal microscopy using the whole film holder with the clamped substrate submerged before injecting certain volumes of an ethanolic dispersion of the CS microgels at the air/water interface, which corresponds to a surface pressure of ≈20 mN m^−1^. The film holder was placed in a sealed stainless‐steel pressure chamber with a quartz glass window for the imaging, as shown in Figure S6B (Supporting Information). The area of interest was illuminated by a cold‐filtered halogen lamp through the reflective light microscope optics and imaged by a color CMOS camera (JAI Go‐2400‐USB, pixel size: 5.86 × 5.86 µm, Stemmer Imaging Puchheim). In combination with the optics (reflected light microscope, extension tube), the resolution of the camera system was 1.72 pixel µm^−1^.

### Methods—Film Thickness During Drying

The drying films with the microgel monolayers started to appear colored due to interference effects when the total film thickness lowered below 1 µm. A model color spectrum was simulated with an algorithm based on a water slab covering a reflective surface (modified from the free‐standing water slab color simulation in Refs. [22, 70]). The spectrum was represented in the hue, saturation, and value (HSV) color space, and was stored in thickness steps of 1 nm in a lookup table. Each film thickness in the range from 100 to 1000 nm has a corresponding set of unambiguous HSV values. Pixel by pixel the hue values of the film image were automatically compared with the lookup table and the corresponding film thickness for each pixel was the result. The height of their shell's shoulder (*H*, Figure 9) was traced as the thin fluid layer dries. For each image and each particle, *H* was calculated using the average color of a circular region around the core, stemming from thin film interference (note that the areas occupied by the cores themselves were excluded from the analysis).

### Methods—Particle Tracking

The detection of the onset of the XY displacement was achieved via particle tracking of the individual microgels in terms of velocity. The averaged velocity over all microgels in the frames does not allow for the precise detection of the onset of the XY displacement because the length and height of the microgel thin film varied in all cases, leading to many frames containing both swollen and collapsed CS microgels in varying ratios. Consequently, ten CS microgels were randomly selected for analysis.

### Methods—Molecular Dynamic (MD) Simulations

The MD simulations were carried out in the canonical (NVT) ensemble using the LAMMPS package.^[^ ^83^ ^]^ In rectangular simulation boxes, 16 identical core‐shell microgels were created near the planar liquid/vapor interface. The monolayers at low and high compression states were prepared and studied. Periodic boundary conditions were applied in xy directions. The system consisted of three types of coarse‐grained beads: liquid/vapor beads (W), solid microgel core beads (C), and polymeric beads (S) forming the microgel shell (Figure S8 and Table S2, Supporting Information). All beads were Lennard–Jones (LJ) particles having the same characteristic size parameter, σ and mass, m. A solid flat wall (sub), referred to as the substrate, was placed at the bottom of the simulation box (z = 0), while a virtual reflective wall was introduced at the top (z = Lz).

The Lennard–Jones truncated and shifted (LJTS) fluid model described via 12–6 LJTS potential was used to simulate the water liquid and vapor phases (details are presented in Supporting Information).^[^ ^84^ ^]^ This highly computationally efficient model accurately describes simple fluids, enabling us to introduce and study the liquid/vapor interface. The vapor‐liquid interfacial properties of the model were extensively investigated in the literature.^[^ ^85^, ^86^, ^87^, ^88^ ^]^ In our model, ε_*W* − *W*_ = 1ε, was applied at *T* = 0.72ε/*k_B_*.

The substrate was modeled via 12–6 LJTS potential as a smooth, solid, flat wall at the bottom of the simulation box (details are presented in Supporting Information). Such “simplification” of the substrate description was reasonable, keeping in mind that the roughness scale of the clean glass surface (hydrophilic) as well as of PFOES coating (hydrophobic) was negligibly small compared to the size of the microgel. To capture the specific hydrophobicity or hydrophilicity of the substrate, the intrinsic contact angle θ of a water droplet was estimated by variation in the interaction strength parameter ε_*W* − *sub*_. It was found that ε_*W* − *sub*_ =  1.7 ε and 3.8 ε accurately reproduce intrinsic contact angles of water droplets of ≈100° and 3°, representing hydrophobic and hydrophilic substrates, respectively.

The core‐shell structure of the microgels was designed using the methodology described in our previous work^[^ ^89^ ^]^ and detailed in the Supporting Information. In analogy with the experiment, the microgels were designed with a more crosslinked inner part near the solid core and a fuzzy outer shell. The average fraction of crosslinkers in the polymeric shells equals 9.4% (see Table S3, Supporting Information). The polymeric beads (S) – liquid interaction was estimated based on the diagram of states of the droplet, which contains polymer, as obtained in Ref. [90]. The combination of parameters ε_*S* − *S*_, ε_*W* − *S*_ with a fixed ε_*W* − *W*_, controls the swelling and localization of the microgels. At the temperature of interest, ε_*S* − *S*_ =  0.275ε,  ε_*W* − *S*_ = 0.5ε ensures that the microgels were in a swollen state in bulk and a collapsed state after drying while also positioning the microgel at the liquid‐vapor interface.

To simulate solvent evaporation and the drying of soft interfacial films onto a solid substrate, a deletion zone with a thickness of 10σ was introduced at the top of the simulation box (z = Lz). The evaporation rate was controlled by adjusting the number of vapor particles removed from the deletion zone and the frequency of their removal (details are presented in Supporting Information).

## Conflict of Interest

The authors declare no conflict of interest.

## Supporting information



Supporting Information

Supplemental Video 1

Supplemental Video 2

Supplemental Video 3

Supplemental Video 4

## Data Availability

The data that support the findings of this study are available from the corresponding author upon reasonable request.
