# Supplementary material for: Drying of Soft Colloidal Films
Source: Adv Sci (Weinh). 2024 Nov 5;11(47):2406977. doi: 10.1002/advs.202406977 (PMC11653679; doi:10.1002/advs.202406977)
Supplement: Supplementary file 1 — Supporting Information [file ADVS-11-2406977-s002.pdf]

ADVANCED SCIENCE
Open Access

Supporting Information

for *Adv. Sci.*, DOI 10.1002/advs.202406977

Drying of Soft Colloidal Films

*Keumkyung Kuk, Julian Ringling, Kevin Gräff, Sebastian Hänsch, Virginia Carrasco-Fadanelli, Andrey A. Rudov, Igor I. Potemkin, Regine von Klitzing, Ivo Buttinoni and Matthias Karg**

Supporting Information

Drying of Soft Colloidal Films

Keumkyung Kuk,[a] *Julian Ringling,*[a] *Kevin Gräff,*[b] *Sebastian Hänsch,*[c] *Virginia Carrasco-Fadanelli,*[d] *Andrey A. Rudov,*[e,f] *Igor I. Potemkin,*[e,f] *Regine von Klitzing,*[b] *Ivo Buttinoni,*[d] *and Matthias Karg*[a]*

[a]Institut für Physikalische Chemie I: Kolloide und Nanooptik, Heinrich-Heine-Universität Düsseldorf, Universitätsstr. 1, 40225 Düsseldorf, Germany

[b]Institute for Condensed Matter Physics, Soft Matter at Interfaces, Technische Universität Darmstadt, Hochschulstr. 8, 64289 Darmstadt, Germany

[c]Center for Advanced Imaging, Heinrich-Heine-Universität Düsseldorf, Universitätsstr. 1, 40225 Düsseldorf, Germany

[d]Institut für Experimentelle Physik der kondensierten Materie, Heinrich-Heine-Universität Düsseldorf, Universitätsstr. 1, 40225 Düsseldorf, Germany

[e]Leibniz Institute for Interactive Materials, 52056 Aachen, Germany

[f]Physics Department, Lomonosov Moscow State University, Moscow 119991, Russian Federation

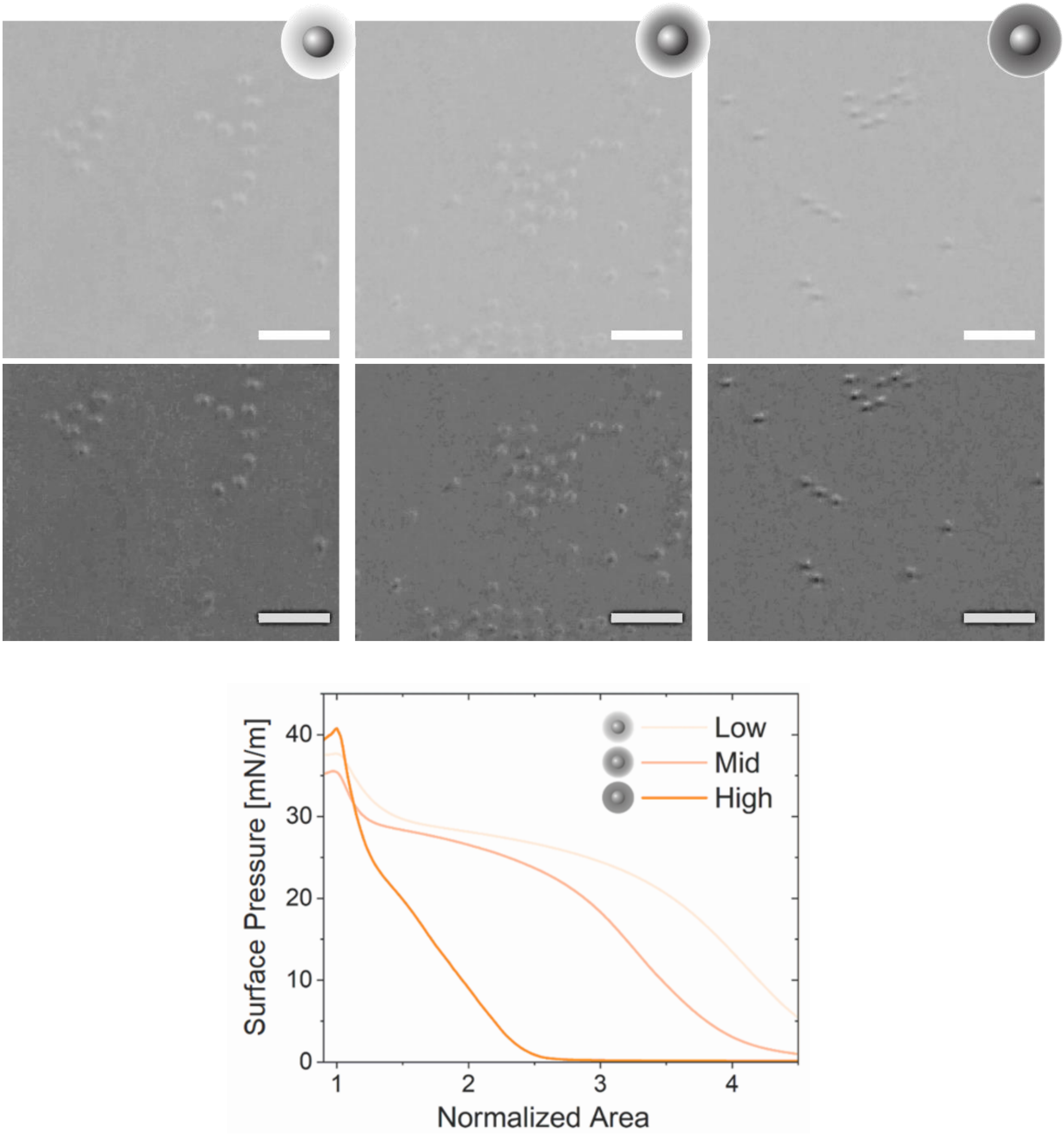

Figure S1. Top row: Light microscopy images (in reflection) of CS-low (left), CS-medium (middle), and CS-high (right) at the air/water interface at a surface pressure of approx. 0 mN/m. At least 200 microgels were used for the acquisition of the mean nearest center-to-center distance, $D_{c\text{-}c}$, in **Figure 1** in the main manuscript. Bottom row: Parts of the images shown in the top row with adjusted brightness and contrast to better visualize the microgels. The scale bars correspond to 5 µm. The graph below shows the compression isotherms of the corresponding microgels.

Table S1. The critical height, H^*, of CS microgels at the water/solid interface and deformation rate at the air/water interface.

	Water/solid interface				Air/water interface	
	Hydrophilic		Hydrophobic		*Ethanolic	**Aqueous
	H^* [nm]	H^*/D_h	H^* [nm]	H^*/D_h	Degree of deformation (D_i/D_h)	
CS-low	159 ± 35	15.8%	157 ± 14	15.6%	170.5%	174.5%
CS-medium	301 ± 6	27.0%	169 ± 33	15.1%	133.7%	136.4%
CS-high	511 ± 6	48.6%	409 ± 23	38.8%	109.2%	109.2%

*adsorption to the air/water interface from ethanolic dispersion (ethanol as the spreading agent) and **from aqueous dispersion (spontaneous adsorption). The measurements were done after 30 minutes of equilibration time for more than 200 microgels at 0 mN/m.

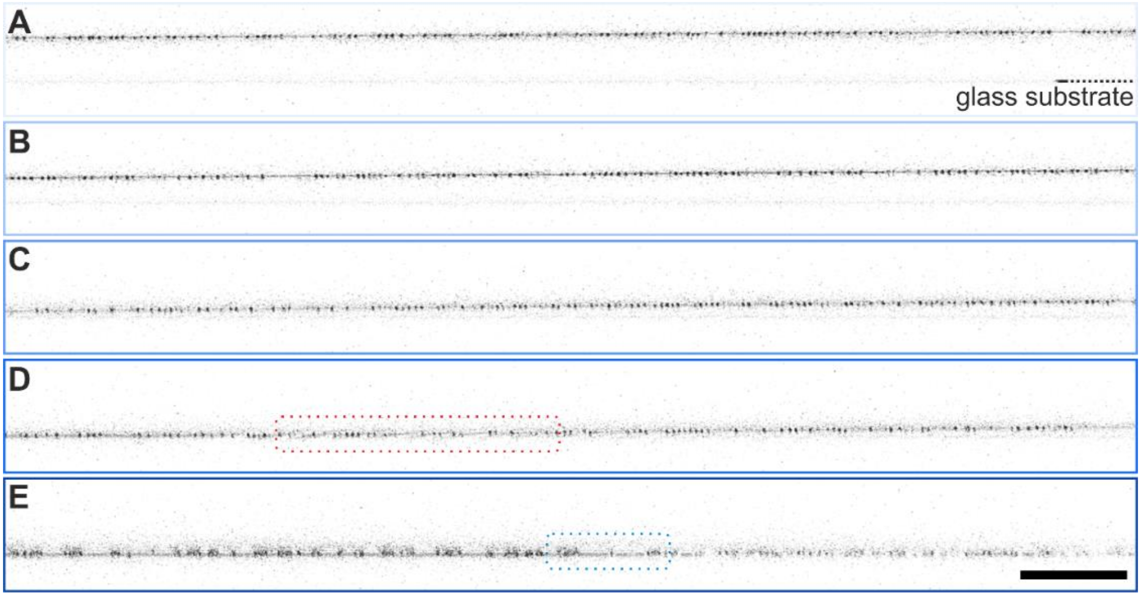

Figure S2. In situ monitoring of the drying CS-medium film on the hydrophilic substrates via confocal microscopy. **A-C** depict the lowering of the CS-medium monolayer (upper line, dots are rhodamine B dyed core) to the substrate (lower line). **D** shows the interference pattern that arises from the slope of the meniscus (receding wetting angles θ_1, in the red box with a dotted line) and the monolayer thin film on the left. **E** shows the drying front of the microgel monolayer indicated by the higher intensity of the dried monolayer. The scale bar corresponds to 20 μm.

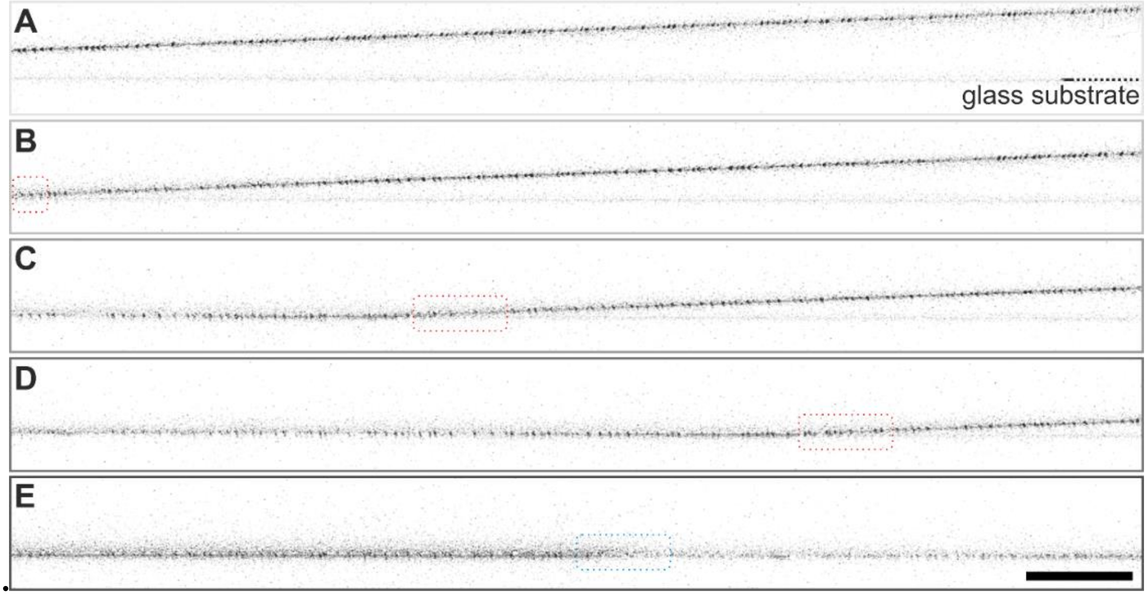

Figure S3. In situ monitoring of the drying CS-medium film on the hydrophobic substrate via confocal microscopy. **A** and **B** depict the lowering of the CS-medium monolayer to the substrate. **B**-**D** show the beginning of the monolayer thin film and the receding wetting angles, θ_2. **E** shows the drying front of the microgel monolayer indicated by the higher intensity of the dried monolayer. The scale bar corresponds to 20 μm.

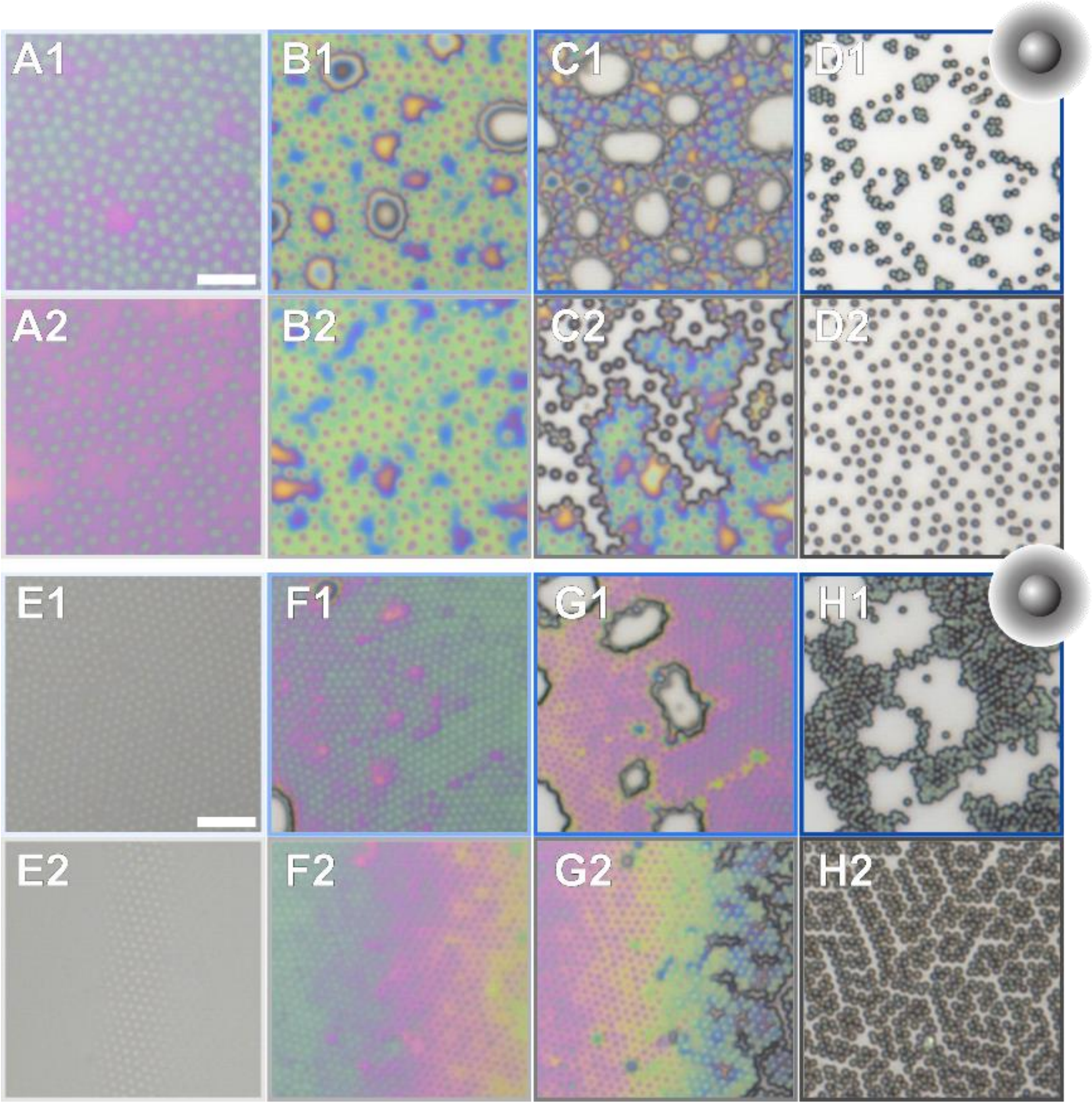

Figure S4. In situ monitoring of the drying of CS-medium film, transferred from Π near 10 mN/m, drying on hydrophilic (**A1**-**D1**) and on hydrophobic substrates (**A2**-**D2**). (**E1**-**H2**) are the same data sets for the microgel film transferred from Π near 30 mN/m. The scale bars correspond to 5 μm.

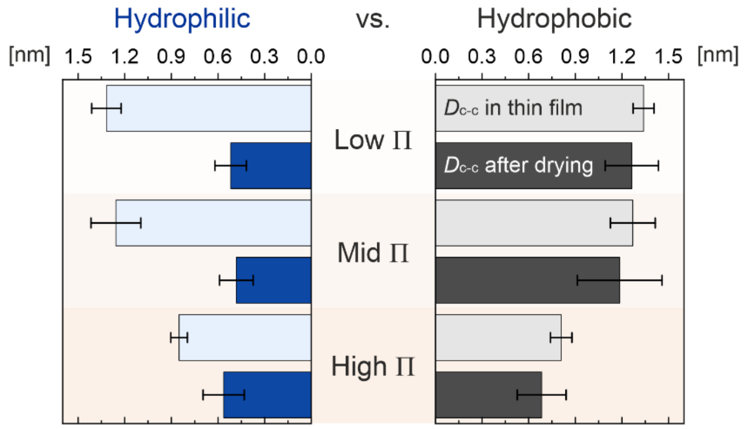

Figure S5. Center-to-center distance, $D_{c\text{-}c}$, of CS-medium film drying onto hydrophilic (blue) and hydrophobic (gray) substrates. Distances in thin film are shown using lighter colors and compared to the ones of fully dried microgel clusters (darker colors). The error bars are the standard deviations of the first peaks of the radial distribution functions.

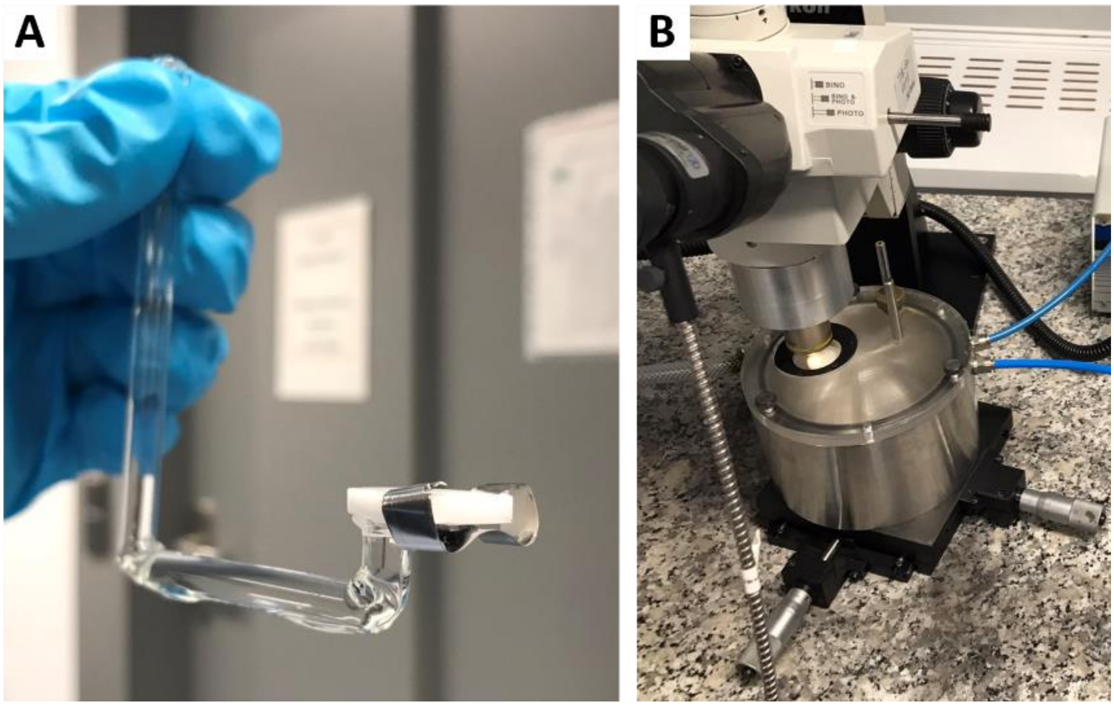

Figure S6. A) A porous glass plate is attached to a glass capillary tube (film holder) used for the thin film pressure balance technique. The sample is attached to the porous glass plate using a stainless-steel clamp. B) The stainless-steel pressure chamber with a quartz glass window is placed under a reflective light microscope.

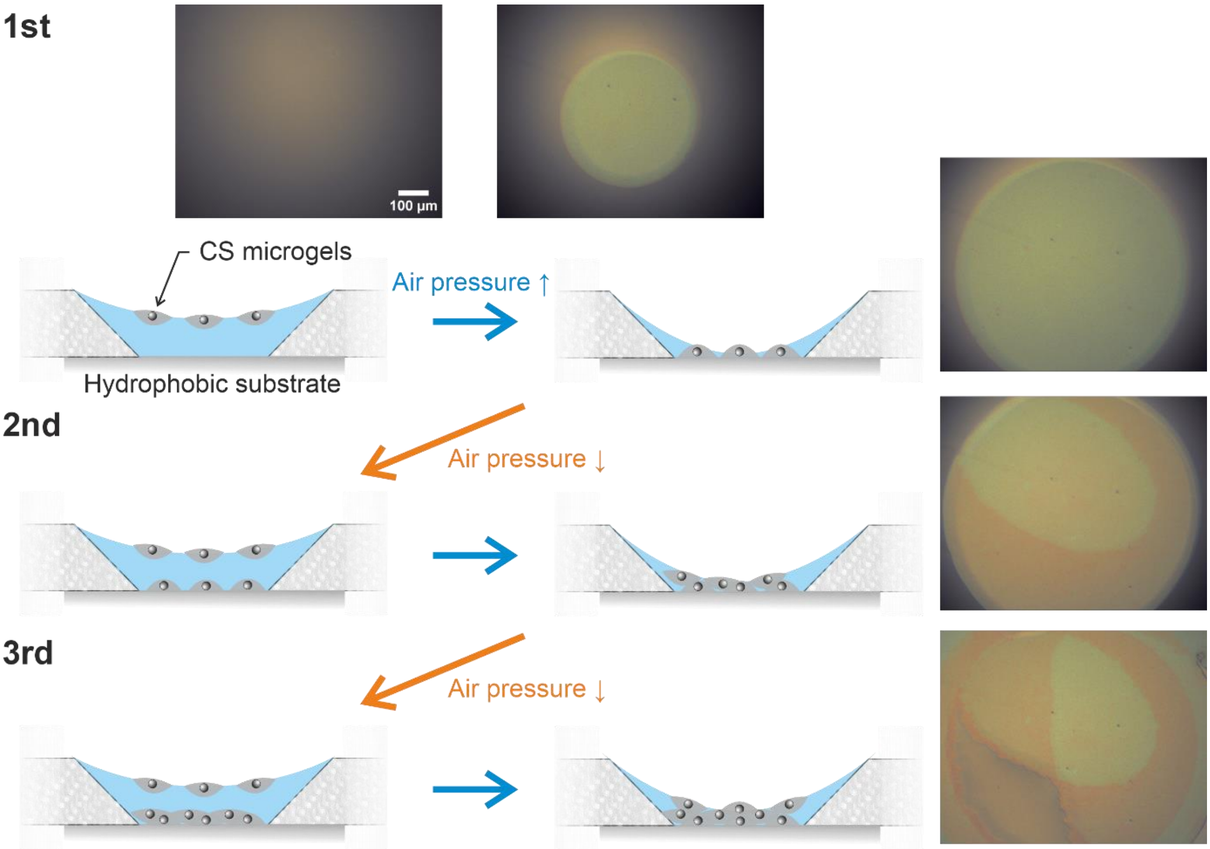

Figure S7. The interface oscillation experiment (3 cycles) using a modified thin film pressure balance setup, depicted in **Figure 3**, only results in the formation of multilayers (3 layers of CS-medium film, indicated by the different colors of the patches) when attempted on hydrophobic substrates.

MODEL AND SIMULATION DETAILS

Molecular dynamics (MD) simulations were conducted using the LAMMPS package [1], employing a standard coarse-grained model with explicit solvent representation. The simulations were carried out in the NVT ensemble in reduced (dimensionless) units derived from the potential parameters, ϵ and σ, mass of a single particle, m, and Boltzmann's constant, k_B. The equations of motions were integrated with a time step, $\Delta t = 0.005\,\tau$, where $\tau = \sigma\,(m/\epsilon)^{0.5}$ is the standard time unit for a Lennard-Jones (LJ) fluid.

Three different types of coarse-grained particles, referred to as beads, are employed in the simulations: water (both liquid and vapor) beads denoted as W, solid microgel core beads designated as C, and microgel polymeric shell beads labeled as S (**Figure S8**, **Table S2**). Each beads have identical mass, m, and size σ.

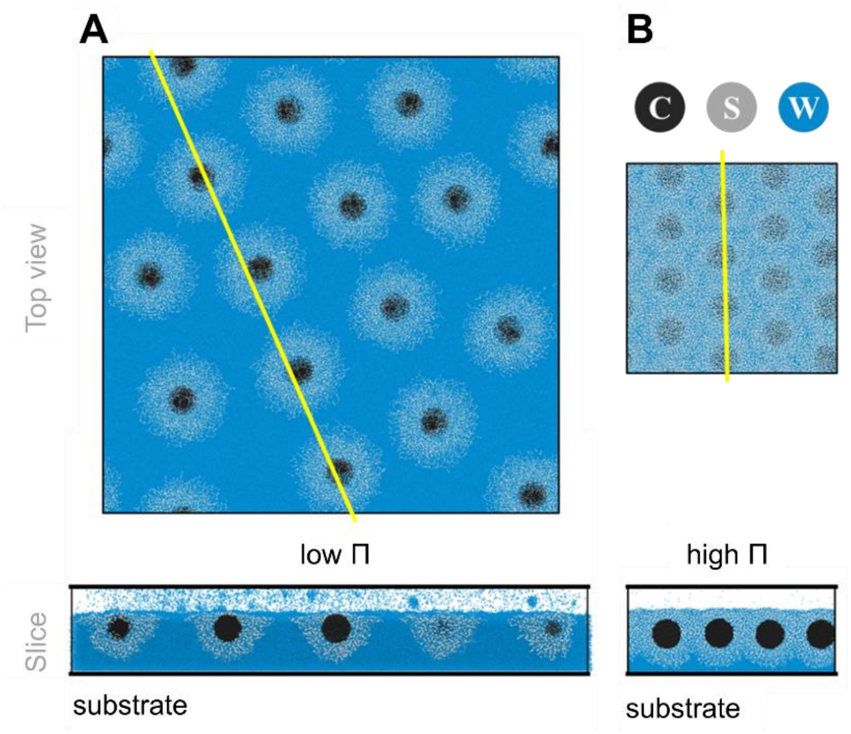

Figure S8. The snapshots of microgel monolayers at liquid/air interface. Cases of low (A) and high (B) compression degrees. View from the air. Solid core, C, shell, S, water, W, and beads are colored black, grey, and blue, respectively. The yellow line indicates the slice area.

Interactions within the system are governed by a standard Lennard-Jones (LJ) 12–6 pairwise potential, characterized by strength ϵ and characteristic length σ:

$$U_{LJ\,smo}(r) = \varphi_{LJ}(r) - \varphi_{LJ}(r_{cut}) \qquad \text{Eq. S1}$$

$$\varphi_{LJ}(r) = \begin{cases} 4\epsilon_{ij}\left(\left(\frac{\sigma}{r}\right)^{12} - \left(\frac{\sigma}{r}\right)^{6}\right), & r \leq r_{cut} \\ 0, & r > r_{cut} \end{cases} \qquad \text{Eq. S2}$$

where $r_{cut} = 3\sigma$, $i, j \in \{W, S, C\}$.

Table S2: MD interaction parameters (in units of ϵ) at $T = 0.72\epsilon/k_B$ used in simulations, where ϵ refers to the LJ energy parameter of the bead-to-bead interaction and k_B is the Boltzmann constant.

	**C**	**S**	**W**	**sub****	**cap****
**C**	0.275	0.275	2*	2*	0.001
**S**		0.275	0.5	ϵ_{S-sub}	0.001
**W**			1	ϵ_{W-sub}	0.001
**sub**				–	–
**cap**					–

*$r_c = 2^{1/6}\sigma$; **$r_c = 3\sigma$;

Liquid–vapor coexistence

We begin by outlining the approach taken to establish the coexistence between the liquid and vapor phases. The system encompasses W beads. The appropriate choice of LJ parameters accurately capturing both bulk and interfacial properties of simple fluids are well described in [2–6]. We set and maintained the system temperature $T = 0.72\ \epsilon/k_B$, using a Nose-Hoover thermostat. The temperature is selected within the range spanning from the triple-point temperature [7], $T = 0.65\ \epsilon/k_B$, to the critical temperature [8], $T = 1.08\ \epsilon/k_B$, thus accurately representing the coexistence of liquid and vapor phases. We defined the interaction parameter between W beads as $\epsilon_{W-W} = 1\epsilon$. Placing W beads uniformly distributed over the empty simulation box eventually leads to the formation of a liquid aqueous phase(droplet) surrounded by water vapor (**Figure S9**). The liquid phase is characterized by a density of $\rho{\sim}0.8/\sigma^3$. The vapor is in dynamic equilibrium with a liquid. Droplet size becomes constant and does not change over time due to the dynamic balance between condensation and evaporation of the W beads.

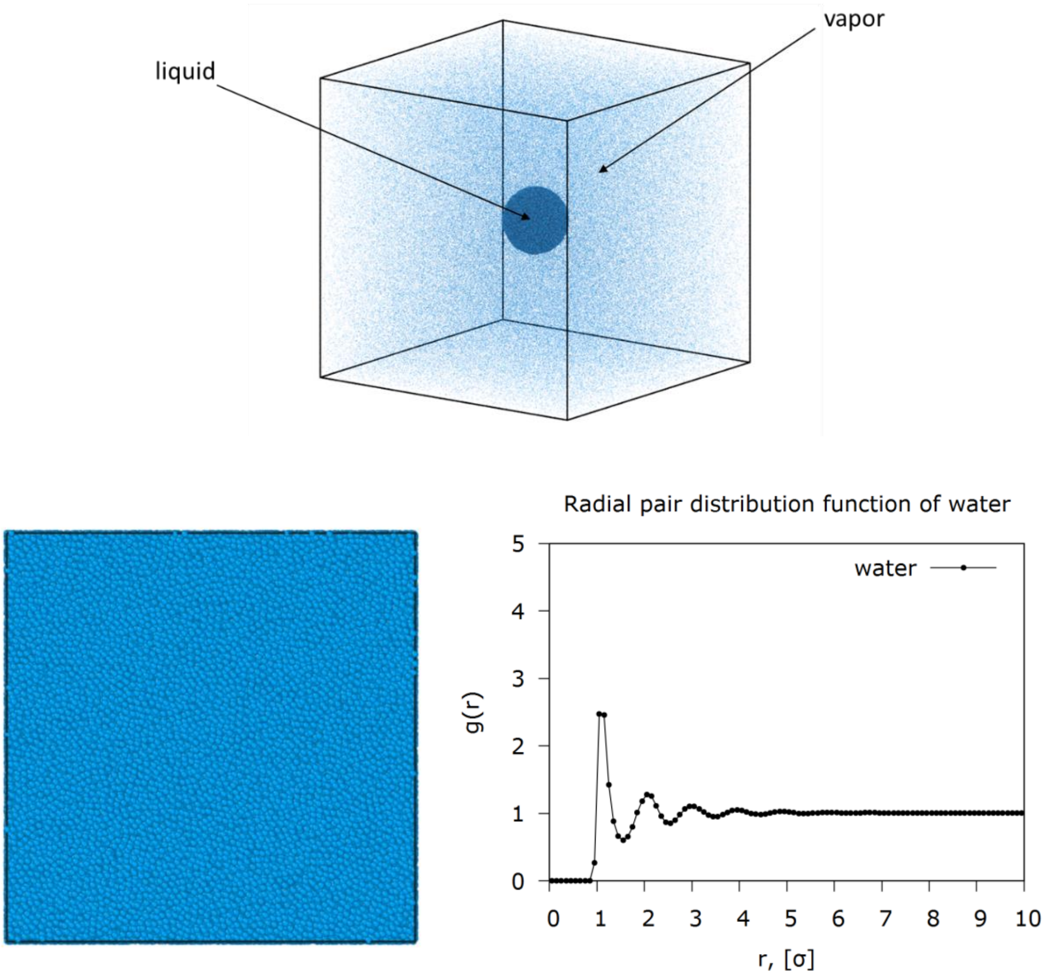

Figure S9: (Top) Liquid-vapor coexistence. (Left) Schematic illustration of the liquid phase. Liquid density $\rho = 0.8/\sigma^3$. (Right) Radial pair distributions function of beads in a liquid phase at $T = 0{,}72\epsilon/k_B$.

Substrate definition

To establish a substrate, we implemented a simplified smooth solid flat wall (sub) represented by an LJ potential (Eq. S2) positioned at the bottom of the simulation box, specifically at z = 0, and functioning as the substrate. This wall exerts a force on each bead in the system in the z-direction, perpendicular to the wall, whenever the distance between the bead and the wall is less than $r_{cut\,wall} = 3\sigma$. Similarly, on the opposite side, at $z = L_z$, we introduced a second wall, serving a technical auxiliary purpose. All interaction parameters between the upper wall and every bead in the system were selected as $\epsilon_{i-top} = 0.001\varepsilon$, $r_c = 2^{1/6}\sigma$, $i \in \{W, C, S\}$ representing complete repulsion. Consequently, we achieved a 2D+1 slab geometry, with periodic boundary conditions exclusively applied in the x and y directions.

This simplification of the substrate description is justified, considering that the roughness of the glass surface and the PFOES coating are much smaller than the size of the microgel. In our study, we focused solely on the effect of surface hydrophobicity/hydrophilicity at a given

temperature. By varying the water-substrate interaction parameter, ϵ_{W-sub}, and microgel-interaction parameter, ϵ_{S-sub}, we could control the substrate's affinity for the liquid and polymer beads, thereby altering the contact angle of the droplet θ (**Figure S10**). It is noteworthy that the intrinsic contact angle θ generally depends on the roughness and density of the substrate [9,10], ρ_s, the LJ energy parameter of the substrate−droplet interaction and the temperature T of the system [11].

To reproduce the specific hydrophobicity/hydrophilicity of the substrate, we determined the intrinsic contact angle θ of a droplet of pure liquid as a function of the interaction strength parameter ϵ_{W-sub}. We placed a spherical water droplet beside the substrate and monitored the shape change. It was found that $\epsilon_{W-sub} = 1.7\epsilon$ and 3.8ϵ correspond to intrinsic contact angles of approximately $\theta \sim 100°$ and ~3° for hydrophobic and hydrophilic substrates, respectively. By adjusting the microgel-substrate interaction parameter, ϵ_{S-sub}, we could regulate the substrate's affinity for the microgels. We explore two distinct shell/substrate interaction scenarios: one with a strong adhesion ($\epsilon_{S-sub} = 20\epsilon \gg \epsilon_{W-sub}$), resulting in significant microgel spreading and the other with a weaker interaction ($\epsilon_{S-sub} = 3\epsilon \sim\epsilon_{W-sub}$), leading to partial wetting.

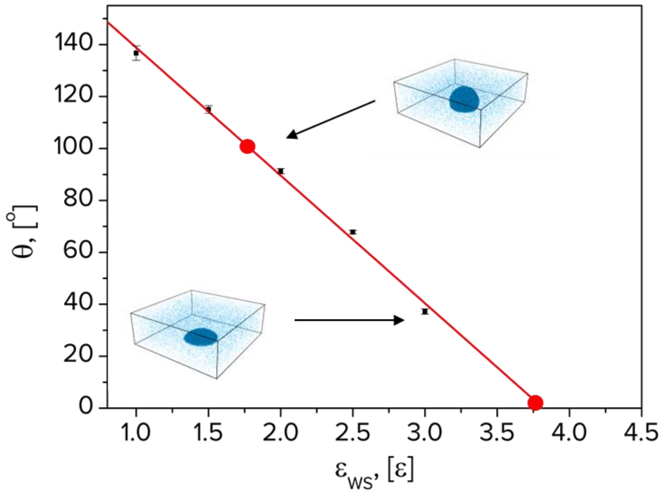

Figure S10: Dependence of contact angle of a water droplet as a function of the interaction energy ϵ_{W-sub} between water and substrate obtained in the computer simulation. For ϵ_{W-sub} values of 1.7ϵ and 3.8ϵ were chosen to obtain the intrinsic contact angles of the pure water at hydrophobic and hydrophilic substrates, respectively.

Microgel overview: model, parametrization, characteristics

CS microgel featuring a hydrodynamic diameter of 40±0.6σ with fuzzy polymeric shells and a spherical solid core of diameter 16σ has been created (**Table S3**). The average fraction of cross-linkers in the polymeric shells equals 9.4%. The core-shell structure was designed following the methodology outlined in our prior works. Initially, a unit cell of the diamond crystal lattice was created, with tetrafunctional cross-linkers at the vertices. Subsequently, two cubic

supercells denoted as S50 and S25, were constructed. S50, comprised of 50×50×50 unit cells, serves as the template for the solid nanoparticle, while S25, consisting of 25×25×25 unit cells, acts as the template for the polymeric shell.

A solid nanoparticle was constructed by inscribing a spherical frame into an S50 supercell and removing all beads outside the sphere. The beads composing the nanoparticle are labeled as C.

Table S3: Characteristics of the core-shell microgel with an anisotropic distribution of cross-links.

**Sample**	Core	Inner shell				Outer shell				Shell(Total)	
	**N_{beads}**	**N_{beads}**	**$N_{cross\text{-}links}$**	$\bar{l}$	σ_l	**N_{beads}**	**$N_{cross\text{-}links}$**	$\bar{l}$	σ_l	**N_{total}**	**% cross-links**
**CS**	3020	4342	778	3	1	12654	810	6	1	16996	9.4

In line with experimental findings, a simulation was conducted to replicate the structure of core-shell microgels, featuring an inhomogeneous cross-link distribution across their inner and outer regions. This was achieved by constructing a polymeric shell around the solid nanoparticle using a scaled S25 supercell. Within this shell, all bonds between tetrafunctional atoms were substituted with polymer subchains of varying lengths. The length of these subchains dictated the degree of cross-linking within the microgel: shorter subchains corresponded to higher cross-link densities, and vice versa. Different laws can describe the distribution of the chain lengths. We will use a symmetric Gaussian distribution with an average value of $\bar{l}$, and a standard deviation of σ_l. $\sigma_l = 0$ corresponds to the case of an ideal diamond-like network used in many simulations. The higher the σ_l the stronger the discrepancy of the network from the ideal regular structure. Then, three spherical frames of different radii were inscribed into the modified supercell. A small frame is necessary for forming the void in the polymeric network of the same size as the solid nanoparticle. The sizes of the middle, R_M, and large, R_L, frames control the thickness of the polymeric shell's inner and outer regions, respectively. All beads were cropped inside the small and outside of the large ellipsoidal frames. The rest of the beads forming the microgel shell were denoted as S. Pair of $(\bar{l}_{in}, \sigma_{in})$ and $(\bar{l}_{out}, \sigma_{out})$ set the distribution of the cross-linkers within the regions. Such a procedure allows us to synthesize polymeric shells with highly cross-linked inner parts and gradually decrease cross-linker concentration towards the outer part. The solid nanoparticle was inserted into the void of the microgel with further grafting of the dangling chains of the polymeric shell to the nanoparticle surface. The dangling chains of the polymeric shell were physically attached to the nanoparticle surface. Going through all of the free ends of the dangling chains, we monitor whether the distance between it and the closest bead at the surface of the solid core satisfies the condition: $|r_{end} - r_{surf}| \leq r_c$. If so, a bond between the free end of the dangling chains and the bead of the surface of the solid core was formed. Only one bond could be formed between the free end and the solid core.

Connectivity of the beads into a polymer network was maintained by a combination of the finite extension nonlinear elastic (FENE) potential and Lennard-Jones potential:

$$U_{bond}(r) = U_{FENE}(r) + U_{LJ}(r) \quad \text{Eq. S3}$$

where the distance between two beads is denoted by r.

$$U_{FENE}(r) = -\frac{1}{2}KR_{max}{}^{2}\ln\left(1 - \frac{r^2}{R_{max}{}^{2}}\right) \quad \text{Eq. S4}$$

$$U_{LJ}(r) = 4\epsilon_{bond}\left[\left(\frac{\sigma}{r}\right)^{12} - \left(\frac{\sigma}{r}\right)^{6}\right] + \epsilon_{bond} \quad \text{Eq. S5}$$

with the spring constant, $K = 15\ \epsilon/\sigma^2$, the maximum bond length, $R_{max} = 1.5\ \sigma$, $\epsilon_{bond} = 1\epsilon$, and the cutoff radius $r_{cut} = 2^{1/6}\ \sigma$.

The angle potential, which is necessary to maintain the spherical shape of the solid core and prevent it from deformation at the interfaces, is given by

$$U_{angle} = K_a(\theta - \theta_{0,}), \quad \text{Eq. S6}$$

where $K_a = 100\epsilon$ is the bending stiffness, and $\theta_0 = 109.47°$ is the angle between two pairs of connected C beads sharing a common bead.

To mimic the swollen state of the microgel in the bulk solution, we set the value of the LJ parameter for the microgel bead-to-bead interactions to $\epsilon_{S-S} = 0.275\epsilon$. For the interactions between the shell and water, $\epsilon_{S-W} = 0.5\epsilon$ was chosen. The idea behind these is as follows: at $\epsilon_{S-W} = 0.5\epsilon$ and $\epsilon_{S-S} = 0.275$ all subchains of the microgel in the bulk are elongated, providing maximum swelling. At the same time, in the absence of solvent (after the complete evaporation of the liquid), the polymer shell collapses. The values for the core-water interaction $\epsilon_{C-W} = 2\epsilon, r_c = 2^{1/6}\sigma$ are chosen to be purely repulsive to prevent flocculation and ensure the insolubility of particles.

Microgel monolayer formation at the air/liquid interface

A planar liquid/vapor interface was established and aligned parallel to the xy plane. In proximity to the interface, 16 microgels with identical architectures were randomly distributed. The degree of compression was controlled by concurrently adjusting the dimensions of the simulation box in both the X and Y directions. For low Π values, the simulation cell was configured as a rectangular box with dimensions $L_x = L_y = 260\sigma, L_z = 50\sigma$. Conversely, for high Π values, the dimensions were adjusted to $L_x = L_y = 120\sigma, L_z = 50\sigma$. **Figure S8** provides an overview of the monolayers of CS microgels at two different compression states – low (A) and high Π (B). Similar to [12], the microgel at the interface is a non-flat object. It consists of a three-dimensional swollen part, namely the bulky part (B-part), immersed in the liquid phase, and a thin two-dimensional polymeric layer, namely the substrate part (S-part), at the interface. At low Π (**Figure S8A**), $L_x = L_y = 260\sigma$, some of the microgels start to come into contact with each other. According to [12] the microgel surface coverage is attributed to the region of the first growth on the compression curve, where the 2D S-parts of the microgels start to be in contact with each other. We performed a qualitative analysis of the monolayer. We calculated the center of mass of each particle and computed the average 2D radial distribution functions, g(r). Examples of the 2D Voronoi tessellation of the simulation box and representative g(r) are shown in **Figure S11**. The position of their first maximum of g(r) allows

us to estimate inter-particle distance, $D_{\text{c-c}}$ = 50.1± 0.4σ. (**Table S4**). At $L_x = L_y = 120\sigma$, the surface pressure of the monolayers proceeds to the second increase (high Π value). The 2D shell-to-shell contacts are replaced by 3D interactions of the B-part of the microgels (**Figure S8B**). $D_{\text{c-c}}$ = 31.2± 0.2σ.

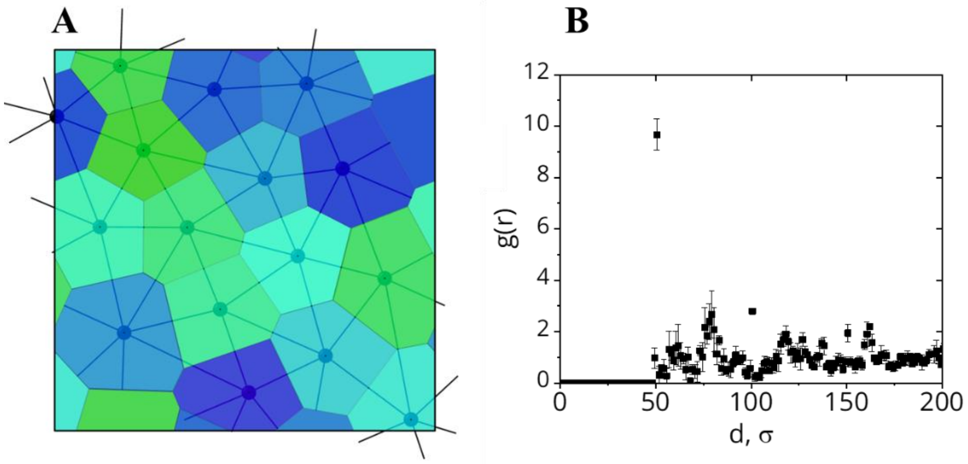

Figure S11. (A) 2D Voronoi tessellation of the simulation box, taking the center of mass of the solid core particles as Voronoi cell centers. (B) The radial pair distribution function, RDF, is for the center of mass of the solid core particles. Cases of low compression degree. Low surface affinity to microgels.

Drying dynamics and structural changes of microgel monolayer

We aim to compare monolayers transferred directly onto both hydrophilic and hydrophobic substrates. Following an equilibration run of 10^7 timesteps at the air/liquid interface, we initiate evaporation (**Figure S12A**). This involves introducing a deletion zone with a thickness of 10σ at the top of the simulation box, positioned at $z = L_z$. Within this zone, water vapor beads are consistently removed from the system to modulate the balance between condensation and evaporation. The evaporation rate is regulated by the number of particles removed and the frequency of removal. Specifically, every 0.5τ, all solvent beads were extracted, emulating rapid solvent evaporation in a vacuum. This setup allows us to explore the scenario wherein monolayer adsorption proceeds concurrently with evaporation.

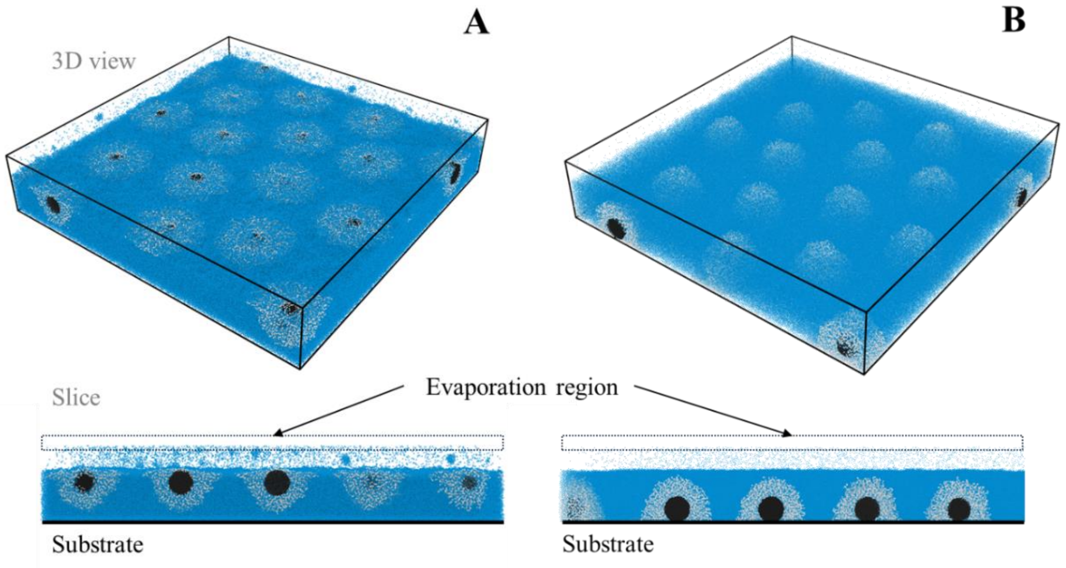

Figure S12: (A) Equilibrium structure of monolayer of microgels near the solvent/air interface. (B) The microgels' monolayer structure is equilibrated in a liquid phase near the substrate.

Figure 5 presents the time evolution of the monolayers (obtained at low Π) during drying. Utilizing an orange-to-pink color gradient, we visualize variations in water layer height across distinct film regions. This dynamic phenomenon encompasses solvent redistribution, microgel shape and size changes, and morphological shifts within the monolayer.

It is noteworthy that the evaporation process within the monolayer exhibits non-uniformity. Notably, rapid evaporation occurs predominantly in the spaces between the microgels, where the absence of the polymeric network facilitates efficient vapor escape. Conversely, the network-like structure of the microgels restricts evaporation within their vicinity. This inherent asymmetry initiates the development of a drying front within the polymeric-free voids, prompting the displacement of adjacent microgels toward the drying direction. Consequently, we observe a discernible migration of the microgels from their initial positions. To regulate the affinity of the microgels to the substrate, we varied the parameter ϵ_{S-sub} within the range [3ϵ, 20ϵ]. A higher ϵ_{S-sub}, corresponds to a stronger attraction between the substrate and the microgels. It is evident that even at $\epsilon_{S-sub} = 20\epsilon$ the microgel-to-substrate adhesion is insufficient to prevent XY displacement. Nevertheless, the mobility of the microgels on the hydrophobic substrate is impeded. The higher the affinity, the less susceptible the microgels are to displacement. This is corroborated by a higher $D_{c\text{-}c}$, value and the distribution of the microgels in the dried layer, as detailed in **Table S4**.

In the context of solvent evaporation, microgels undergo significant shape and size alterations. As they dry, microgels tend to collapse, albeit with varying degrees of spreading. Microgel-substrate contacts are advantageous on hydrophobic surfaces, prompting microgels to displace solvent underneath, thereby increasing contact area with the substrate (**Figure 4C**), leading to

microgel spreading. Conversely, hydrophilic surfaces favor contact with water, resulting in a minimal contact area with the substrate (**Figure 4B**). This interplay culminates in the spontaneous displacement of microgels and the disruption of the observed monolayer morphology at the water/air interface under the given surface pressure (Π).

Table S4. Interparticle distance, $D_{c\text{-}c}$, of dried CS monolayers on hydrophilic substrates and hydrophobic substrates in a thin film, as compared to that at Air/water interphase. ϵ_{S-sub} is the affinity of the microgels to the substrate. $\epsilon_{S-sub} = 20\epsilon$ corresponds to the case of high affinity, while $\epsilon_{S-sub} = 3\epsilon$ – low one.

	**Low Π**		**High Π**	
	hydrophilic $D_{c\text{-}c}$, [σ]	hydrophobic $D_{c\text{-}c}$, [σ]	hydrophilic $D_{c\text{-}c}$, [σ]	hydrophobic $D_{c\text{-}c}$, [σ]
$\epsilon_{S-sub} = 20\epsilon$	36.2± 0.3	38.1± 0.2	29.7± 0.2	29.8± 0.2
$\epsilon_{S-sub} = 10\epsilon$	34.4± 0.2	35.6± 0.2	29.7± 0.3	29.6± 0.2
$\epsilon_{S-sub} = 3\epsilon$	28.3± 0.2	28.9± 0.2	29.8± 0.3	29.7± 0.3
Air/water	50.1± 0.4		31.2± 0.2	

In the case of the film obtained under high compression (Π), as the drying process ensues, the film thickness diminishes, accompanied by a slight collapse of the microgels, as depicted in **Figure S13**,**14**. $D_{c\text{-}c}$ value fluctuates around 29.7σ, a measure akin to the $D_{c\text{-}c}$ of the dry film at low Π, demonstrating good agreement with experimental observations. In contrast, on the hydrophilic surface, we did not observe any deviation from the initial position of the microgels. This lack of deviation can be attributed to the influence of periodic boundary conditions with a constant number of gels, ensuring system closure. It is crucial to note that in a real experiment, the system is open after the gels are transferred to the substrate, leading to a variation in the number of gels per surface unit. Due to their already collapsed state and minimal surface interactions, the microgels demonstrate heightened mobility, facilitating facile migration.

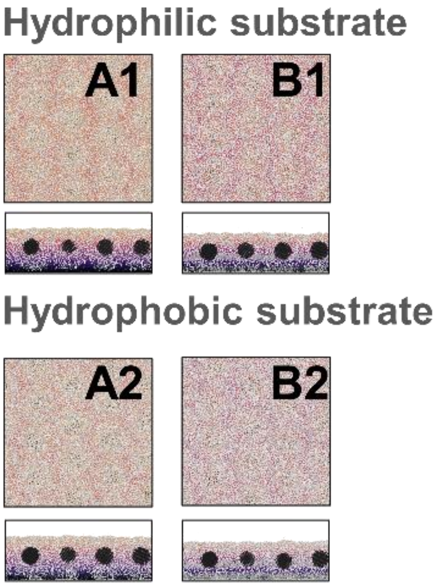

Figure S13. Illustration (top views) of the different stages of microgel film obtained at high Π values drying on hydrophilic (A1,B1) and hydrophobic (A2,B2) substrates as revealed by computer simulations. The narrow panels below the top views are the corresponding side views, i.e., cross-sections of each panel along Z. The height of the film with residual water is depicted by the vertical color bar. White regions in the snapshots correspond to state after water evaporation.

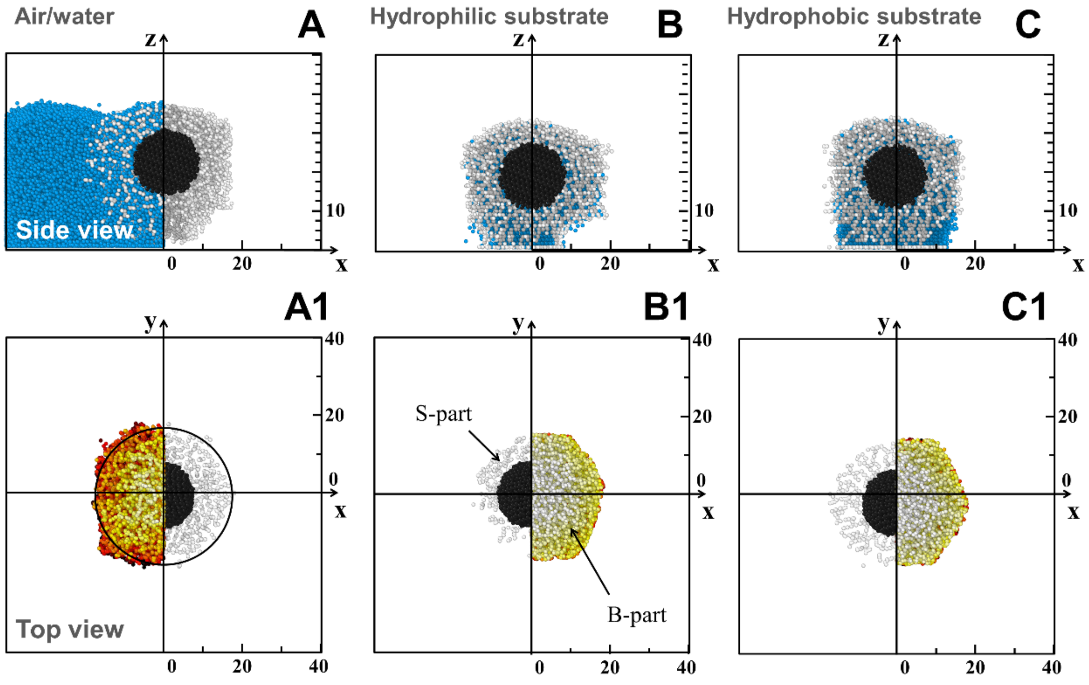

Figure S14. Illustration of the allocated microgel structure in the monolayer before (A-A1) and after drying in the case of hydrophilic (B-B1) and hydrophobic (A2-E2) substrates. The monolayer was transferred at a high compression degree. (A1-C1) Height map / Contact area at the interface. All snapshots are obtained using the Open Visualization Tool (OVITO) [13].

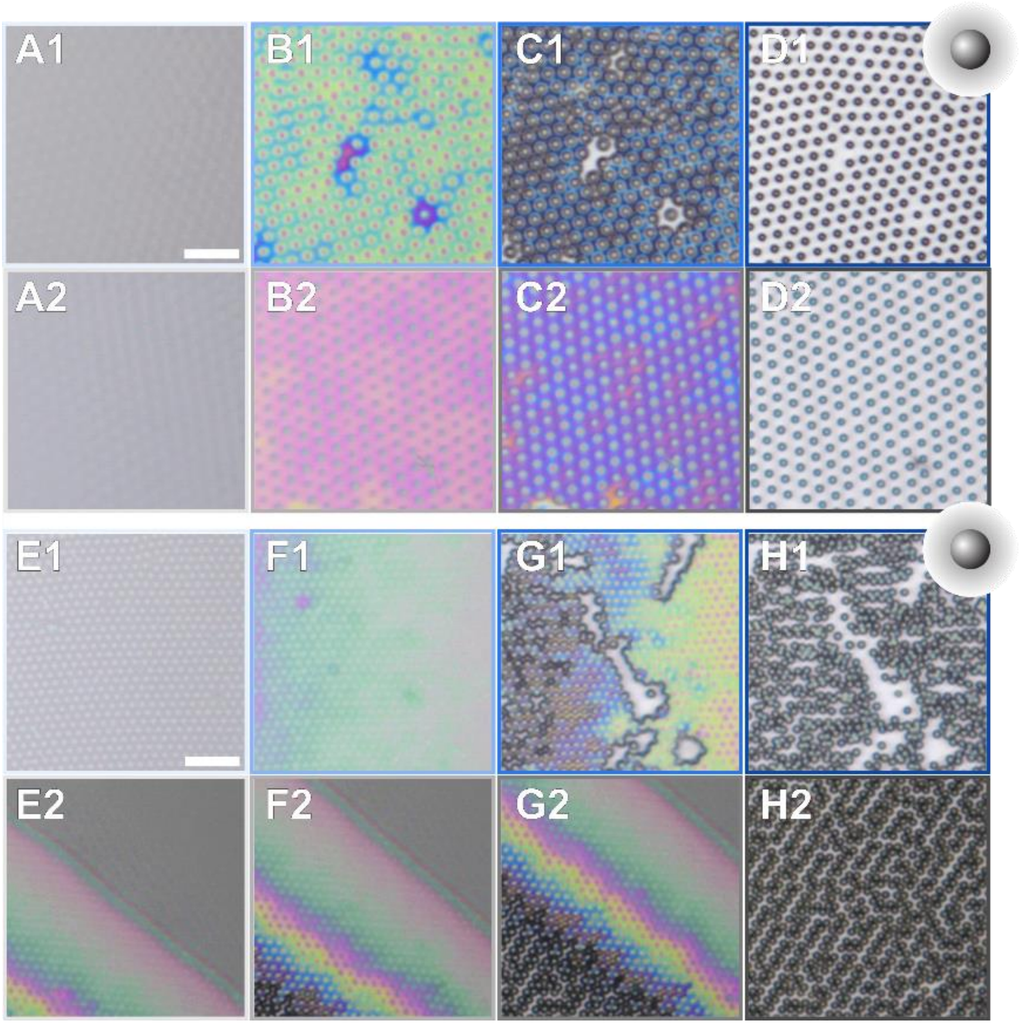

Figure S15. In situ monitoring of the monolayer of CS-low transferred from Π near 10 mN/m, drying on hydrophilic (**A1**-**D1**) and on hydrophobic substrates (**A2**-**D2**). (**E1**-**H2**) are the same sets of data for the monolayer transferred from Π near 30 mN/m. The scale bars correspond to 5 μm.

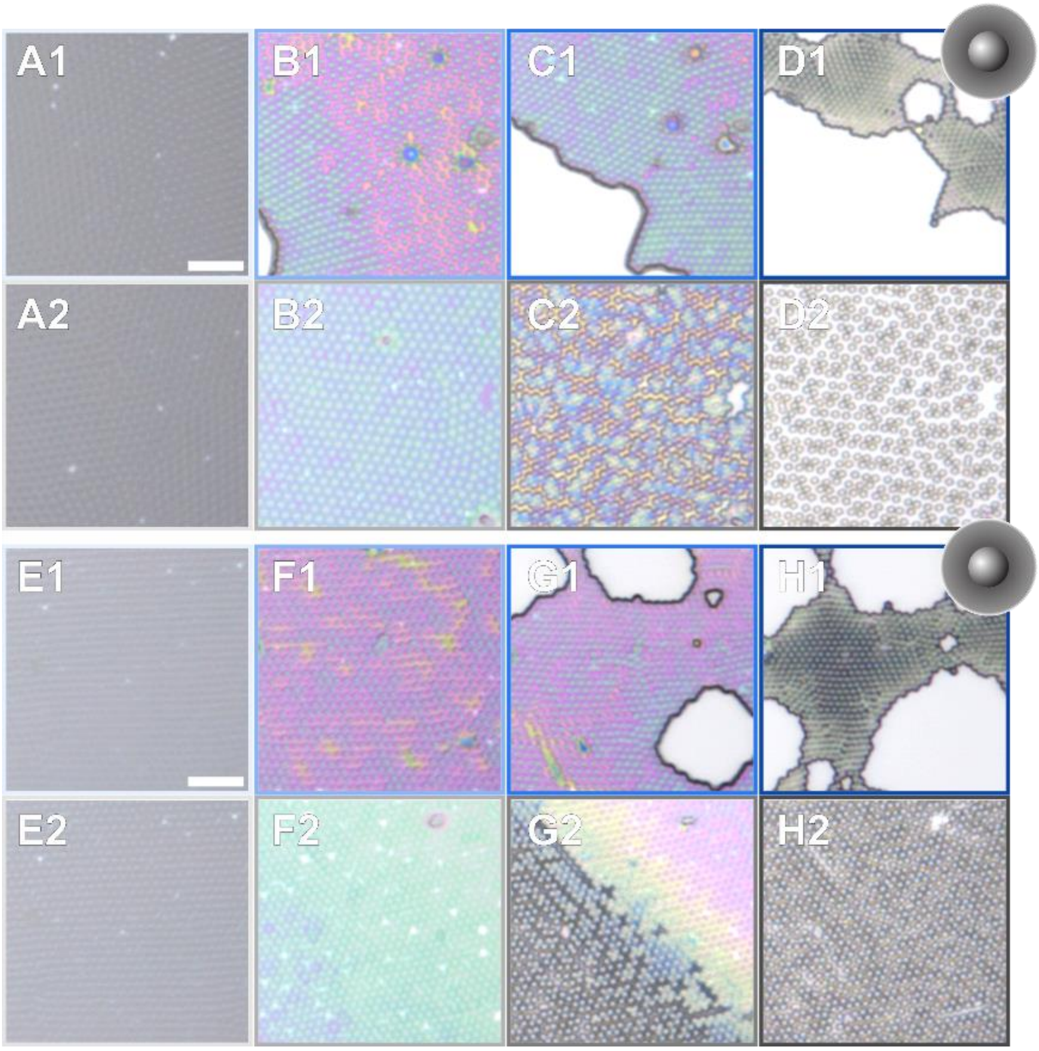

Figure S16. In situ monitoring of the monolayer of CS-high transferred from Π near 10 mN/m, drying on hydrophilic (**A1**-**D1**) and on hydrophobic substrates (**A2**-**D2**). (**E1**-**H2**) are the same sets of data for the monolayer transferred from Π near 30 mN/m. The scale bars correspond to 5 μm.

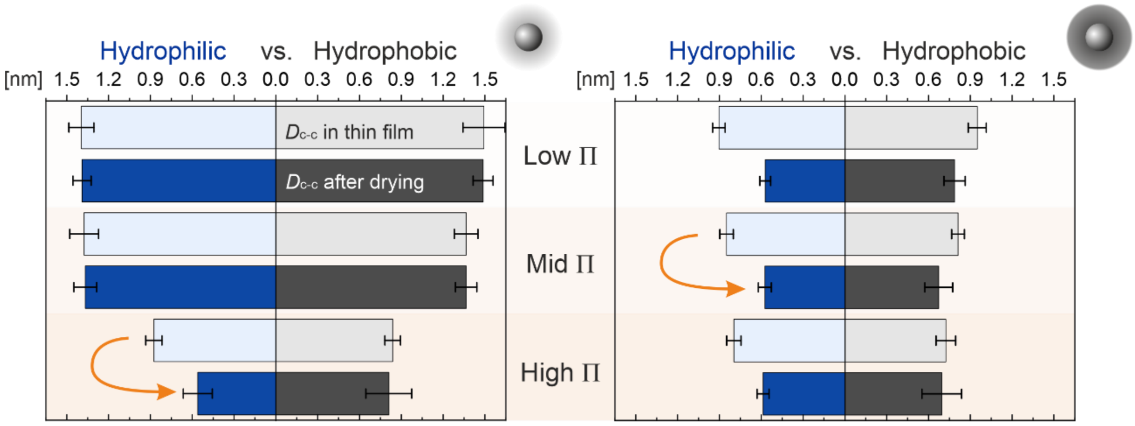

Figure S17. Center-to-center distance, $D_{c\text{-}c}$, of CS-low and CS-high. Films drying on hydrophilic (blue) and hydrophobic substrates (gray) in the thin fluid film (lighter color), as compared to that of dried monolayer (darker color).

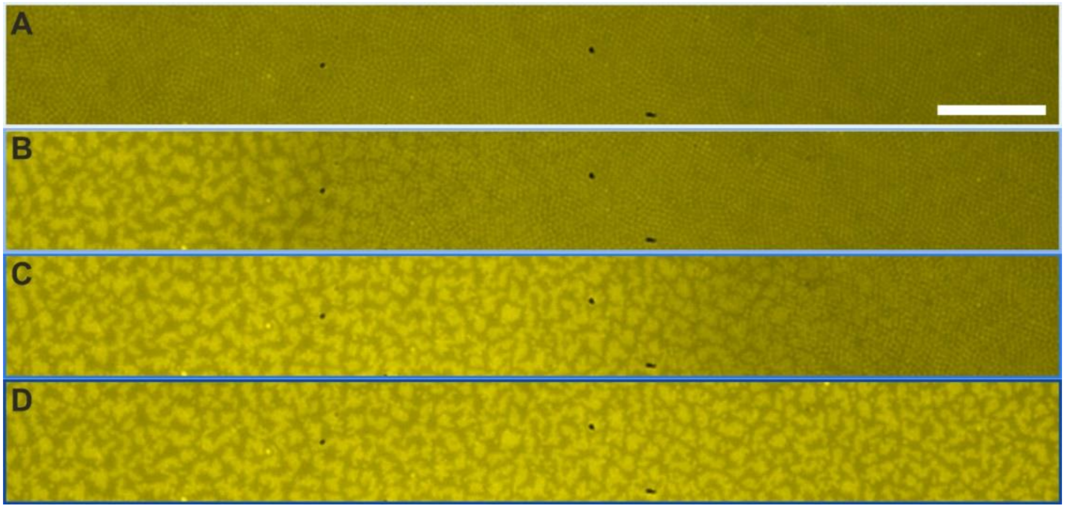

Figure S18. Drying of coreless PNIPAM microgel (sMG, $D_h \approx 800$ nm) film on a hydrophilic substrate via fluorescence microscopy, scale bar corresponds to 20 μm.

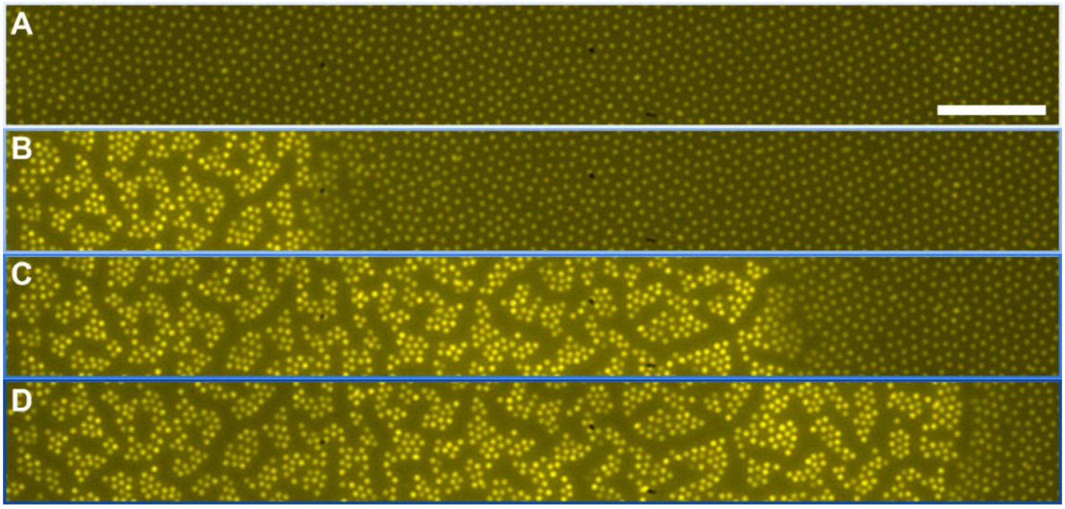

Figure S19. Drying of coreless PNIPAM microgel (lMG, $D_h \approx 1.5$ μm) film on a hydrophilic substrate via fluorescence microscopy, scale bar corresponds to 20 μm.

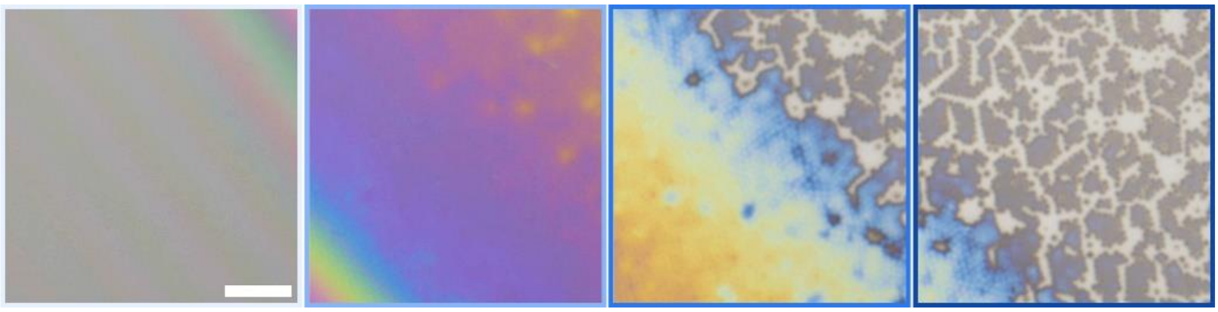

Figure S20. Drying of a small CS microgel (D_c: 105 nm D_h: 504 nm, cross-linker density: 5 mol.%) film on a hydrophilic substrate, transferred from Π approx. 30 mN/m, scale bar corresponds to 5 μm.

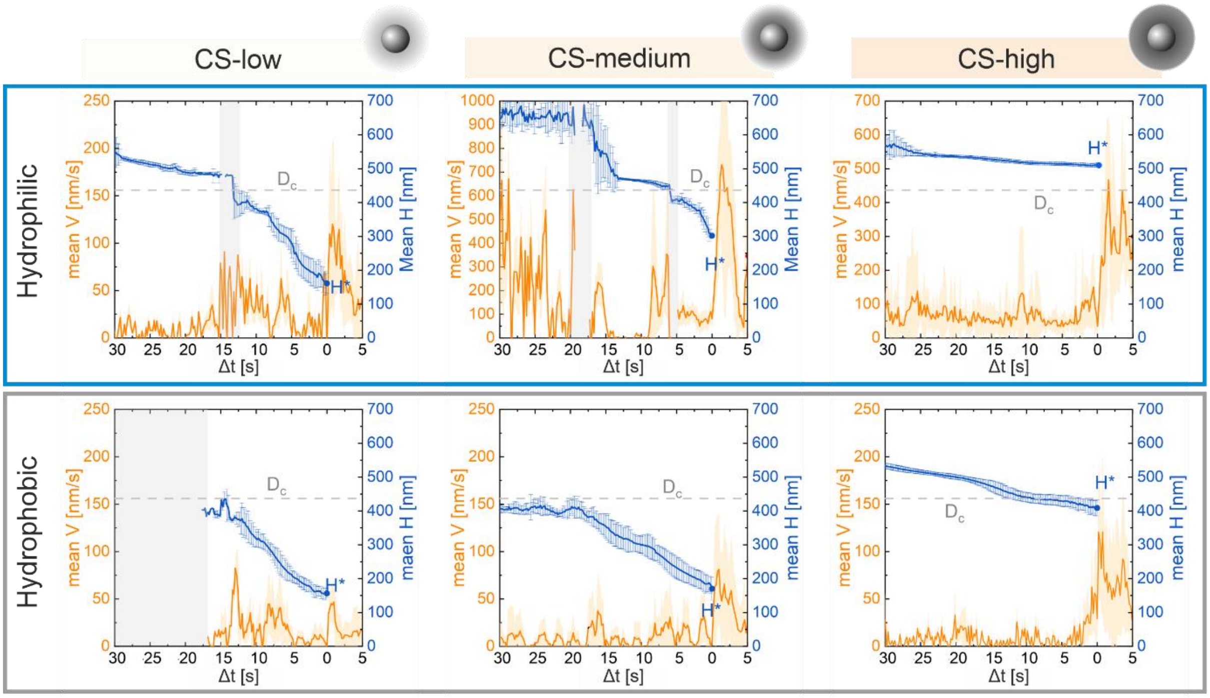

Figure S21. The mean velocity, *V*, and film height, *H*, of 10 randomly chosen CS microgels are plotted against normalized time, where 0 is the onset of the collapse of the individual microgels. D_c and H^* denote the diameter of the core and the critical height of the monolayer, respectively. The shadowed areas (grey) are frames where the monolayers were out-of-focus. Monolayer drift with preferred directions is baseline-subtracted.

Supporting Literature

[1] S. Plimton, Journal of Computational Physics 1995, 117, 1.

[2] W. Chen, J. Koplik, I. Kretzschmar, Phys. Rev. E 2013, 87, 052404.

[3] M. Svoboda, A. Malijevský, M. Lísal, J. Chem. Phys. 2015, 143, 104701.

[4] J. Zhang, F. Leroy, and F. Müller-Plathe, *Evaporation of Nanodroplets on Heated Substrates: A Molecular Dynamics Simulation Study*, Langmuir **29**, 9770 (2013).

[5] A. E. A. S. Evangelopoulos, A. N. Rissanou, E. Glynos, I. A. Bitsanis, S. H. Anastasiadis, V. Koutsos, Macromolecules 2018, 51, 2805.

[6] S. N. Sun,H. M. Urbassek, J. Phys. Chem. B 2011, 115, 13280.

[7] J. A. van Meel, A. J. Page, R. P. Sear, D. Frenkel, J. Chem. Phys. 2008, 129, 204505.

[8] J. Vrabec, G. K. Kedia, G. Fuchs, H. Hasse, Mol. Phys. 2006, 104, 1509.

[9] X. Yong, L. T. Zhang, Langmuir 2009, 25, 5045.

[10] B. A. Noble, B. Raeymaekers, Langmuir 2019, 35, 8784.

[11] S. Becker, H. M. Urbassek, M. Horsch, H. Hasse, Langmuir 2014, 30, 13606.

[12] S. Bochenek, A. A. Rudov, T. Sassmann, I. I. Potemkin, W. Richtering, Langmuir 2023, 39, 18354 .

[13] A. Stukowski, Model Simul. Mat. Sci. Eng. 2010, 18, 015012.
